# Central memory-enriched Vγ9Vδ2 γδ T cells via TGF-β expansion demonstrate enhanced *in vivo* efficacy against metastatic osteosarcoma

**DOI:** 10.3389/fimmu.2025.1657760

**Published:** 2025-09-03

**Authors:** Jordan A. Silva, Kokila Gunasinghe, Hunter C. Jonus, Gianna M. Branella, Austre Y. Schiaffino Bustamante, Jennifer Okalova, Jason T. Yustein, H. Trent Spencer

**Affiliations:** ^1^ Cancer Biology Program, Graduate Division of Biological and Biomedical Sciences, Emory University, Atlanta, GA, United States; ^2^ Aflac Cancer and Blood Disorders Center, Department of Pediatrics, Emory University School of Medicine and Children’s Healthcare of Atlanta, Atlanta, GA, United States; ^3^ Molecular Systems Pharmacology Program, Graduate Division of Biological and Biomedical Sciences, Emory University, Atlanta, GA, United States

**Keywords:** gamma delta (γδ) T cells, osteosarcoma, TGF-β, innate immunotherapy, expansion

## Abstract

The application of cellular immunotherapies (CI) for osteosarcoma (OS) has mainly focused on autologous products of αβ T cells and, to date, has shown little clinical benefit. Based on the multi-killing properties of γδ T cells, specifically Vγ9Vδ2 T cells, and their ability to be employed as an allogeneic, off-the-shelf cellular therapy, there is significant interest in this CI. Although there are efficient, clinical-scale expansion protocols, a concern is the short *in vivo* half-life of these cells due to the terminal differentiated phenotypes of expanded cells. Therefore, modifying the manufacturing process to generate a more memory-like phenotype could overcome hurdles associated with this CI. Transforming growth factor-beta (TGF-β) is a cytokine with multiple functions, and can induce a less differentiated phenotype in γδ T cells. We tested the hypothesis that the *in vivo* effectiveness of γδ T cells against osteosarcoma (OS) tumors is suboptimal because of the manufacturing process that produces terminally differentiated cells. We combined a modified expansion process with activation strategies known to enhance γδ T cell-based tumor killing. Introducing zoledronate (ZOL) to OS cells augments γδ T cell killing by upregulating phosphoantigens in treated cells, which induces butyrophilin complexes, which are recognized by the TCR of the γδ T cell and significantly increases target cell death in both control and TGF-β expanded γδ T cells. In addition, administering ifosfamide (IFO), a chemotherapy used for relapsed OS, induces stress antigens in OS cell lines that are recognized by NKG2D receptors on γδ T cells, which enhances γδ T cell killing. *In vivo* studies show the administration of TGF-β expanded γδ T cells, when combined with ZOL and IFO significantly increased overall survival in OS-bearing mice, which we show can be attributed, at least in part, to increased persistence compared to control cells. Together, these data demonstrate this chemoimmunotherapy strategy, which engages various targeting mechanisms of γδ T cells, significantly enhances killing of OS.

## Introduction

Osteosarcoma (OS) is among the deadliest pediatric cancers with a survival rate of 27% in high-risk, distal, or Stage 4, patients (1). OS is the most common primary malignant pediatric bone tumor with a worldwide incidence of 3.4 per million people (2). Approximately 1 in 5 OS patients at first diagnosis have metastatic disease or are at the distal stage (1). The standard-of-care for OS requires a complex, multidisciplinary regimen for treatment, involving chemotherapy and surgery (3). However, in recent years, immunotherapy-based clinical trials for OS have dramatically increased (4). Current immunotherapies and clinical trials for OS include, but are not limited to, vaccines and dendritic cells, cytokines, checkpoint inhibitors, adoptive cell therapy (ACT), CAR T cells and CAR macrophages, and combination therapies using antibodies, like anti-GD2, also known as Dinutuximab(NCT05634369, NCT05726383, NCT05400603) (5, 6). While these immunotherapies enhance some immune activity against OS, to date, none have changed the course of primary treatment, resulting in no significant change in survival rates over the past 50 years (7–9).

Developing efficacious immunotherapies for OS has been challenging, as this malignancy is a low-immunogenic and heterogenous tumor with a variety of genetic profiles (7, 8).

Unlike adaptive, αβ T cells, which have been the choice product for ACT, γδ T cells, have both potent adaptive immunity and innate-like characteristics (10). In humans, γδ T cells are primarily classified into two major subsets based on their Vδ chain usage. Vδ1 T cells are predominantly found in the thymus and peripheral tissues, where their TCR is thought to recognize MICA/MICB and CD1c/CD1d (11, 12). In contrast, Vδ2 T cells represent the majority of γδ T cells circulating in the blood and their TCR recognizes the accumulation of phosphoantigens in target cells (13–18). These innate-like characteristics allow γδ T cells to have an intrinsic ability to induce cytotoxicity against tumors and cancer cells in their non-modified form (19). This ability comes from the cell surface receptors that γδ T cells express (i.e. γδ-TCR, NKG2D, DNAM-1) and their capability to recognize antigens independently of HLA (20). HLA-independence also allows for γδ T cells to be used in an allogeneic setting with minimal risk of graft-versus-host disease, enabling the production of off-the-shelf, donor-derived γδ T cell therapies (21).

However, a concern regarding the use of γδ T cells, particularly Vγ9Vδ2 T cells, is the lack of persistence. Transforming growth factor (TGF) β is a cytokine with pleiotropic effects on adaptive immunity, especially on the regulation of αβ and γδ T cells (22, 23). The effects of TGF-β are context dependent, and it is becoming evident that TGF-β enhances Vγ9Vδ2 T cell immunotherapy (22, 24), possibly by extending the *in vivo* half-life of these cells.

Additionally, interleukin (IL)-15 shares many functions similar to IL-2, such as regulating innate, as well as adaptive, immune responses (25). The addition of IL-15 to the expansion process of γδ T cells has been shown to enhance their proliferation, stimulatory phenotype, antitumor effector functions, and survival (26–29). In fact, Fowler et al., engineered γδ T cells to secrete a synthetic IL-15 fusion protein that demonstrated enhanced cytotoxicity by activating both direct tumor lysis and bystander immune responses, without the need for exogenous cytokine support (29). The addition of both TGF-β and IL-15 in the expansion process of γδ T cells has been shown to enhance cytotoxic activity, but the memory phenotypes and *in vivo* capabilities have not been well characterized (24), especially in the context of OS.

In the context of neuroblastoma (NB), a highly aggressive pediatric solid tumor, standard therapies such as chemotherapy, surgery, and anti-GD2 immunotherapy, have improved outcomes, but relapsed and refractory cases remain largely incurable (30). Preclinical and clinical data have shown chemoimmunotherapy regimens combining dinutuximab with chemotherapy agents (e.g., irinotecan and temozolomide) sensitize tumors to immune cell mediated killing while reducing immunosuppressive barriers (31, 32). These studies, along with data demonstrating the robust *in vitro* and *in vivo* cytotoxicity of expanded γδ T cells against NB, provided the rationale for initiating our first-in-human Phase I clinical trial against relapsed/refractory neuroblastoma and osteosarcoma (NCT05400603). This trial represents the translational culmination of multiple studies dissecting the cellular components of γδ T cell expansions and tests their therapeutic potential in a clinical setting.

While there are multiple ways of expanding γδ T cells (22, 29, 33–35), our previous studies established an optimized *ex vivo* Vγ9Vδ2 T cell expansion protocol, showed that non-modified γδ T cells are cytotoxic against various cancers, and genetically modifying γδ T cells with chimeric antigen receptors can be an effective therapeutic against both solid and liquid tumors (31, 36–43). Here, we show that our chemoimmunotherapy approach using γδ T cells expanded under various manufacturing conditions, combined with zoledronate, a bisphosphonate that sensitizes osteosarcoma cells to γδ T cells, and ifosfamide, a chemotherapy used for relapsed OS, is a safe and effective method of inducing OS cell death and prolongs *in vivo* survival.

## Materials and methods

### Cancer cell lines

The 143B and U2-OS cell lines were kindly gifted by the Laboratory of Dr. Kelly Goldsmith (Emory University, Atlanta, GA, USA) where they were verified to be free of mycoplasma contamination using the MycoAlert contamination kit (Lonza). The 143B-Luciferase tagged cell line was kindly gifted by the Laboratory of Dr. Jason T. Yustein (Emory University, Atlanta, GA, USA) where they were verified to be free of mycoplasma contamination using the MycoAlert contamination kit (Lonza). The IMR5 cell line was cultured in RPMI-1640 with L-glutamine (Corning), supplemented with 10% fetal bovine serum (FBS; R&D Systems) and 1% Penicillin-Streptomycin (Cytiva). The 143B, 143B-Luc, U2-OS, and MG-63 cell lines were cultured in DMEM (Corning) supplemented with 10% FBS and 1% Penicillin-Streptomycin. All cell lines were cultured at 37 °C in a 5% CO_2_ incubator.

### NKG2D ligand expression on healthy donor and patient samples

The R2 Genomics Analysis and Visualization Platform (https://r2.amc.nl) was used to query multiple datasets using the Megasampler R2 module for expression of ULBP1 and MICA/MICB by RNA sequencing. Exclusively human datasets were queried, and the u133p2 chip with MAS5.0 normalization was used. Datasets utilized in this study were as follows: Tumor OS (Kobayashi 27) and Normal Tissue/Cells (Tsunoda 24).

### Expansion of γδ T cell

γδ T cell expansions were performed based on our previously published technique (31, 36–38, 40–42, 44). PBMCs were procured from healthy adult volunteers who consented to donate whole blood through Emory University’s Children’s Clinical and Translational Discovery Core (CTDC) under the core’s IRB approved protocol (IRB00101797). All participants in this study were under 40 years old and self-reported to be in good physical condition. To isolate PBMCs, approximately 40 mL of whole blood was layered onto Ficoll-Paque Plus (GE Healthcare Life Sciences) and underwent density centrifugation.

To selectively expand γδ T cells, PBMCs were cultured in CTS™ OpTmizer™ T Cell Expansion medium (Fisher Scientific) supplemented with 1% penicillin/streptomycin and 2 mM L-glutamine. Further expansion studies used either RPMI-1640 with L-glutamine (Corning), supplemented with 10% fetal bovine serum (FBS; R&D Systems) and 1% Penicillin-Streptomycin (Cytiva), TheraPEAK^®^ T-VIVO^®^ Cell Culture Medium (Lonza Bioscience) and 1% Penicillin-Streptomycin (Cytiva), or TexMACS™ GMP Medium (Miltenyi Biotec) and 1% Penicillin-Streptomycin (Cytiva). Cell counts were conducted on days 0, 3, 6, 9, and 12 using a hemocytometer count with trypan blue exclusion to determine viability. Cells were resuspended at a concentration of 1.5 × 10^6^ cells/mL in fresh media every 3 days. Zoledronate (Sigma) at 5 μM and IL-2 (PeproTech) at 500 IU/mL were added to the media on days 0 and 3 of expansion. When specified, IL-15 (PeproTech) at 10ng/mL and TGF-β (BioTechne) at 5ng/mL were additionally supplemented through the entire expansion process every 3 days. On days 6 and 9, IL-2 at 1,000 IU/mL was added. On day 12, γδ T cells were either utilized immediately for experiments or cryopreserved in PlasmaLyte A (Baxter) containing 5% human serum albumin (HSA) and 10% DMSO. Flow cytometry analysis was conducted on days 0, 6, and 12 to validate successful expansion. Successful expansions yielded cultures comprising approximately 80-90% γδ T cells, ≤ 10% natural killer cells and ≤ 10% αβ T cells.

### Expansion immunophenotyping

γδ T cells were characterized for markers of activation, senescence, and exhaustion using flow cytometry. Day 12 expanded cells were washed with FACS buffer (PBS + 2.5% FBS) and immediately following wash, cells were stained with the antibody panel (**Supplementary Table 1**) and eBioscience Fixable Viability Dye eFluor 780 (Thermo Fisher Scientific, Waltham, MA, USA) for 20 min at room temperature. After staining, cells were washed twice with FACS buffer and immediately analyzed for cell surface marker expression on a Cytek Aurora. Data analysis was performed using the FlowJo software (v10).

### Stress antigen expression assays

Osteosarcoma cell lines, 143B and U2OS, were conditioned with either Zoledronate or Palifosfamide, the active metabolite in ifosfamide (IFO), for 24 hours at various concentrations (0 – 200 µM and 0 – 1000 µM, respectively). Cells were detached from the plate using Accutase (Thermo Fisher Scientific, Waltham, MA, USA) and washed with FACS buffer (PBS + 2.5% FBS). Cells were stained with MICA/B (BioLegend, San Diego, CA, USA), ULBP-1 (R&D Systems, Minneapolis, MN, USA), ULBP-2/5/6 (R&D Systems, Minneapolis, MN, USA), TRAIL-R1 (BD Biosciences, Franklin Lakes, NJ, USA), and TRAIL-R2 (R&D Systems, Minneapolis, MN, USA) for 30 mins. Samples were assessed for stress antigen expression by flow cytometric analysis (Cytek Aurora) and analyzed with FlowJo software (v10).

### *In vitro* cytotoxicity assays

To assess the cytotoxicity of γδ T cells against OS cell lines, control or TGF-β expanded cells were co-cultured with 50,000 target cells (143B or U2-OS) stained with VPD450 (BD, Franklin Lakes, NJ, USA) at indicated effector-to-target rations for 4 hours at 37 °C, 5% CO2. When indicated, OS cell lines were preconditioned overnight with 5 µM Zoledronate following VPD450 staining and then washed with PBS before co-cultured with the γδ T cells. Co-cultures were detached from the plate using Accutase (Thermo Fisher Scientific, Waltham, MA, USA) and then washed once with Annexin V Binding Buffer (0.025 mM calcium chloride + 1.4 mM sodium chloride + 0.1 mM HEPES) and stained with 3 μL Annexin V-APC (BioLegend, San Diego, CA, USA) and eBioscience Fixable Viability Dye eFluor 780 (Thermo Fisher Scientific, Waltham, MA, USA) for 20 minutes at room temperature. Cells were then washed once with Annexin V Binding Buffer. If cells were not stained with eBiosciences Fixable Viability Dye eFluor 780, then 7-AAD Viability Dye (BioLegend, San Diego, CA, USA) was added 2 minutes before the sample was subjected to flow cytometric analysis (Cytek Aurora). Samples were analyzed with FlowJo software (v10), where percent cytotoxicity was measured by the sum of either Annexin V+, eFluor 780+, and Annexin V+/eFluor 780+, or Annexin V+, 7-AAD+, and Annexin-V+/7-AAD+ target cells.

### Animal studies

NOD.Cg-Prkdc^scid^Il2rg^tw1Wjl^/SzJ (NSG) mice were purchased from Jackson Laboratories (Bar Harbor, ME, USA) and maintained in a pathogen-free environment at an Emory University Division of Animal Resources facility. All animal studies were conducted in accordance with established policies set forth by the Emory University Institutional Animal Care and Use Committee (IACUC) under an approved animal use protocol (PROTO201800202). Equal numbers of male and female mice were used for all studies.

To test the cytotoxicity of γδ T cells in an OS subcutaneous flank model, 6–10-week-old NSG mice were subcutaneously injected with 5×10^6^ 143B osteosarcoma cells. Tumors were left to grow for ~10 days until tumor volume was between 100-300mm^3^. On day 10, when tumor volume was between 100-300mm^3^, 1×10^7^ γδ T cells (n=3) or 1×10^7^ γδ T cells with 3 µg ZOL (n=3) were injected directly into the tumor, which was shown previously to be an effective route of administration (31). Untreated mice received PBS injections (n=5). Tumor volume and mouse weight were measured three times per week.

To examine the persistence of γδ T cells in mice, 7-13-week-old NSG mice were administered 1×10^7^ non-modified γδ T cells via retro-orbital injection. γδ T cells were expanded under Control conditions (n=4), TGF-β (n=5), TGF-β + IL-15 (n=4), or with TGF-β and then frozen and thawed (n=4). Peripheral blood was collected at 48-, 96-, and 144-hours post-injection and leukocytes were assessed for the presence of human CD45 cells, and then from within that population CD3^+^ and γδ TCR^+^ by flow cytometric analysis (Cytek Aurora) and analyzed with FlowJo software (v10). On day 6, mice were euthanized, and bone marrow was harvested to assess for the presence of human CD45^+^, CD3^+^ and γδ TCR^+^ cells as before.

To assess the cytotoxicity of TGF-β expanded γδ T cells in an OS lung metastasis model, 6–10-week-old NSG mice were intravenously injected via the tail vein with 5×10^5^ luciferase-expressing 143B osteosarcoma cells. The following morning and then once weekly for the next 2 weeks, mice were injected intraperionetially with 200mg/kg IFO (45) and 3ug ZOL (46, 47). Beginning in the afternoon on the next day and then every 3–4 days after for a total of 4 doses, mice were treated with 1×10^7^ Control expanded γδ T cells or TGF-β expanded γδ T cells via retro-orbital injection, with PBS to serve as an untreated control (n=10). Tumor growth and overall health of the mice were monitored two times per week via IVIS (*In Vivo* Imaging System, Revvity, Waltham, MA, USA) imaging and weighing, respectively. IVISbrite D-Luciferin Potassium Salt Bioluminescent Substrate (Revvity, Waltham, MA, USA) was injected at 150mg/kg via intraperitoneal injection 10 minutes prior to imaging. Bioluminescence was quantified using Living Image Software (Revvity, Waltham, MA, USA).

### Statistical analysis

Statistical analyses were performed using GraphPad Prism (v10.0). Results are presented as mean ± standard deviation of the mean and were considered statistically significant at p < 0.05. Unpaired or paired two-tailed Student’s t-test or Mann-Whitney t-test, log-rank (Mantel-Cox) on the Kaplan Meier survival plots, one-way or two-way ANOVA tests, with Tukey’s *post hoc* analysis, were used to determine statistical significance as appropriate. R2 database automatically performed one-way ANOVA when providing results. The results of these statistical analyses can be found in **Supplementary Figure 11**.

## Results

### Zoledronic acid sensitizes osteosarcoma cells to γδ T cell mediated cytotoxicity

**Figure 1:**
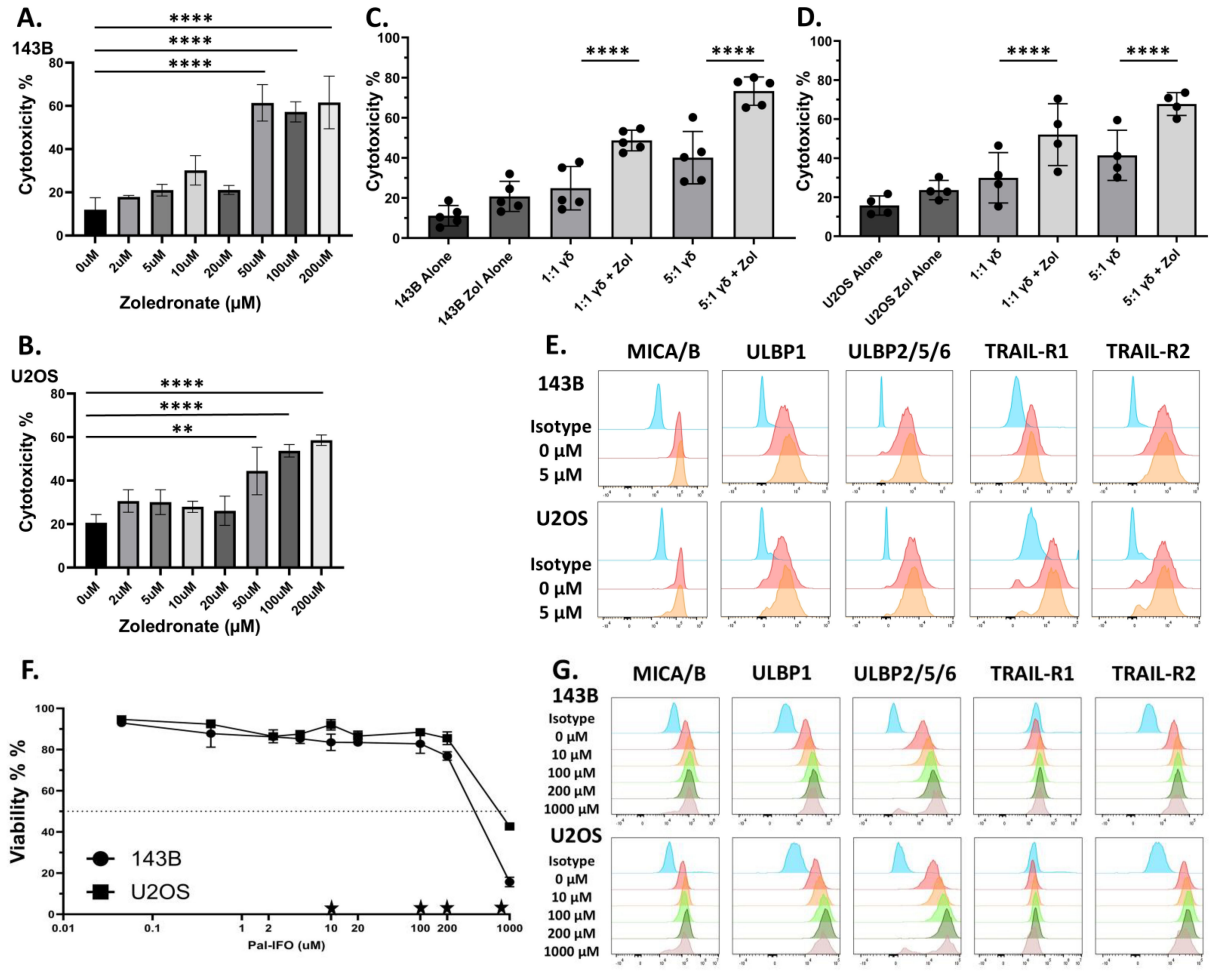
Zoledronate significantly increases non-modified Vγ9Vδ2 γδ T cells cytotoxicity but does not substantially alter stress antigen expression, while ifosfamide increases stress antigen expression. **(A, B)** Osteosarcoma cell lines (**(A)**143B and **(B)** U2OS) were conditioned with various doses of Zoledronate for 24h. Viability was measured via 7AAD and Annexin V. Statistical analysis represents a one-way ANOVA with Tukey’s *post hoc* analysis was done on these data. (** p < 0.01, **** p < 0.0001). Tukey’s statistical analysis can be found in the Supplemental Stats File. Error Bars represent SD. n = 2 experimental replicates. **(C, D)** Osteosarcoma cell lines **(C)** 143B or **(D)** U2OS were conditioned with 5µM Zoledronate for 24h. After 24 h of treatment with 5µM zoledronate or control, osteosarcoma cells were incubated with day 12 *ex vivo*-expanded Vγ9Vδ2 γδ T cells for 4h at 1:1 and 5:1 effector to target ratios (E:T). Viability was measured via 7AAD and Annexin V. Statistical analysis represents a one-way ANOVA with Tukey’s *post hoc* analysis was done on these data. (**** p < 0.0001). Tukey’s statistical analysis can be found in the Supplemental Stats File. Error bars represent SD. n = 4–5 experimental replicates. n = 3 donors **(E)** Flow cytometry histograms display the fluorescent intensity expression of antibodies used for respective stress antigens (MICA/B, ULBP-1, ULBP-2/5/6) and death receptors (TRAIL-R1 and TRAIL-R2) on osteosarcoma cells after conditioning of either control or 5µM zoledronate. Histogram colors from top to bottom: Green = Unstained, Red = Isotype Control, Blue = 0 µM, Orange = 5 µM. Stress ligand expression shown from live cells. Viability measured with eFluor780. n= 2 experimental replicates. **(F)** Dose-response curves showing the viability of osteosarcoma cells (143B and U2OS) after overnight conditioning with Pal-IFO (0-1000 µM). Viability measured with eFluor780. Stars represent doses shown in **(G)**. Error Bars represent SD. n= 2 experimental replicates. **(G)** Flow cytometry histograms display the fluorescent intensity expression of antibodies used for respective stress antigens (ULBP1, ULBP2/5/6, MICA/MICB) and death receptors (TRAIL-R1 and TRAIL-R2) in osteosarcoma cells (143B and U2OS) following overnight treatment with increasing concentrations (0-1000 µM) of Pal-IFO. Stress ligand expression shown from live cells. Viability measured with eFluor780. Histogram colors from top to bottom: Blue = Isotype Control, Red = 0 µM, Orange = 10 µM, Light Green = 100 µM, Dark Green = 200 µM, Grey = 1000 µM. n= 2 experimental replicates.

Zoledronic acid (ZOL) is a bisphosphonate that promotes the intracellular accumulation of phosphoantigens (pAgs), such as isopentenyl pyrophosphate (IPP), by inhibiting farnesyl pyrophosphate synthase, a key enzyme in the mevalonate pathway (**Supplementary Figure 1**). These pAgs are recognized by the intracellular domain of butyrophilin (specifically BTN3A or CD277), which when bound undergo conformational change with BTN2A1 that is specifically detected by the Vγ9Vδ2 γδ TCR (14, 48, 49). To evaluate the direct toxicity effects of ZOL on osteosarcoma, 143B and U2OS osteosarcoma cell lines, were treated with increasing concentrations of ZOL (0-200µM) for 24 hours (**Figures 1A, B**). Both cell lines exhibited dose-dependent increase in cytotoxicity, with minimal effects observed at concentrations below 5µM (p > 0.05) and substantial (p < 0.05) cytotoxicity at 50-200µM (**Figures 1A, B**). Overnight conditioning of 143B and U2OS cells with 5µM ZOL followed by co-culture with γδ T cells led to a significant increase in γδ T cell-mediated killing. This enhanced killing was observed in both 143B and U2OS cell lines at effector-to-target (E:T) ratios of 1:1 and 5:1, which may emphasize the potential role of the γδ TCR-pAg-BTN axis in OS tumor recognition (**Figures 1C, D**).

### Zoledronic acid enhances γδ T cell cytotoxicity independent of NKG2D ligands or death receptor upregulation

To determine whether the observed enhanced cytotoxicity could be driven by stress-induced surface molecule expression on target cells, we also assessed the expression of key activating and death-inducing ligands on osteosarcoma cells following ZOL treatment. Surface molecules such as NKG2D ligands, MHC class I chain-related protein (MIC) family (e.g., MICA and MICB) and the UL16-binding protein (ULBP1-6) family, and the death receptors, TNF-related apoptosis-inducing ligand receptors (TRAIL-R1 and TRAIL-R2), were measured via flow cytometry. The NKG2D ligands, also known as stress antigens, are generally absent or in low amounts on the cell surface of healthy cells but are upregulated when cellular stress occurs. Therefore, in addition to the γδ TCR, NKG2D receptors on γδ T cells can recognize the stress antigens and provide another activation strategy (50, 51). Flow cytometric analysis of ZOL-treated cells confirmed that 5µM ZOL did not statistically upregulate NKG2D ligands or death receptors, suggesting the observed increase in cytotoxicity is not mediated through stress-induced pathways (**Figure 1E**).

### Zoledronic acid enhances γδ T cell anti-tumor activity *In Vivo*

ZOL pre-conditioning enhances γδ T cell mediated cytotoxicity against osteosarcoma cells by promoting γδ TCR-dependent recognition, highlighting the therapeutic potential of phosphoantigen mediated γδ T cell targeting in osteosarcoma (47). To test the *in vivo* therapeutic efficacy of γδ T cells with or without ZOL, a subcutaneous 143B osteosarcoma mouse model was used. As depicted in the experimental schematic, mice were administered 5×10^6^ 143B OS tumor cells subcutaneously and subsequently treated with intratumoral injections of 1×10^7^ γδ T cells on days 10, 13, 17, and 20 (**Supplementary Figure 2A**), which was shown previously to be an effective route of administration in a NB model (31). Tumor volumes were measured three times per week, revealing γδ T cell treatments did reduce tumor growth compared to untreated controls, with the combination of γδ T cells + ZOL producing the greatest reduction in tumor burden (**Supplementary Figure 2B**). Although there was a reduction in the tumor burden in the mice treated with γδ T cells, the reduction was not significant. However, survival analysis demonstrated significantly improved outcomes in mice who received γδ T cells and in the γδ T cells + ZOL group (**Supplementary Figure 2C**). While there was a reduction in tumor burden and a significant increase in survival, these findings were minimal, highlighting the need to develop an improved treatment strategy.

### Palifosfamide enhances stress ligand expression and OS in a dose dependent manner *In Vitro*

Our lab has previously shown the necessity of using chemoimmunotherapy to better enhance the cytotoxic potential of γδ T cells. Therefore, we sought to introduce the chemotherapy ifosfamide (IFO), which is used to treat relapsed OS (45, 52), into our therapeutic regimen. The goal, therefore, is to induce a second activation strategy, which is up-regulation of stress antigens and activation through the NKG2D receptor (53). To assess differential expression of stress-induced ligands in osteosarcoma, we analyzed mRNA levels of NKG2D ligands using publicly available datasets obtained from R2: Genomics Analysis and Visualization Platform (**Supplementary Figures 3A, B**). Both *ULPB1* and *MICA/MICB* transcripts are significantly (p < 0.01) upregulated in osteosarcoma tissues compared to normal tissues, suggesting enhanced baseline stress levels in osteosarcoma tumor cells due to their malignant nature (**Supplementary Figures 3A, B**). IFO undergoes hepatic metabolism facilitated by CYP450 enzymes, generating the active metabolite. Therefore, Palifosfamide was used for *in vitro* assays, as it is the active metabolite of IFO. To determine whether chemotherapeutic conditioning further increases the surface expression of stress ligands in osteosarcoma, 143B and U2OS osteosarcoma cell lines were treated for 12 hours with varying concentrations of Palifosfamide (Pal-IFO, 0-1000 µM), followed by flow cytometric analysis. Both osteosarcoma cell lines exhibited a dose-dependent reduction in viability, with 143B cells appearing to be slightly more sensitive to treatment (**Figure 1F**). A dose-dependent increase in surface expression of ULBP1, ULBP2/5/6, MICA/MICB, TRAIL-R1, and TRAIL-R2 across both cell lines was also observed in response to drug-induced stress (**Figure 1G**). This observation was quantified by measuring mean fluorescence intensity (MFI), which confirmed upregulation of all tested ligands following Pal-IFO conditioning, particularly at higher concentrations, in both 143B and U2OS cell lines (**Supplementary Figures 3C, D**). These findings support the potential of IFO as a dual-function agent that i) has direct cytotoxic impact on OS cells and ii) could potentially sensitize osteosarcoma cells to immune-mediated killing, particularly by innate-like effector cells such as γδ T cells.

### TGF-β drives expansion of central memory-enriched, non-terminally differentiated γδ T cells in various media

**Figure 2:**
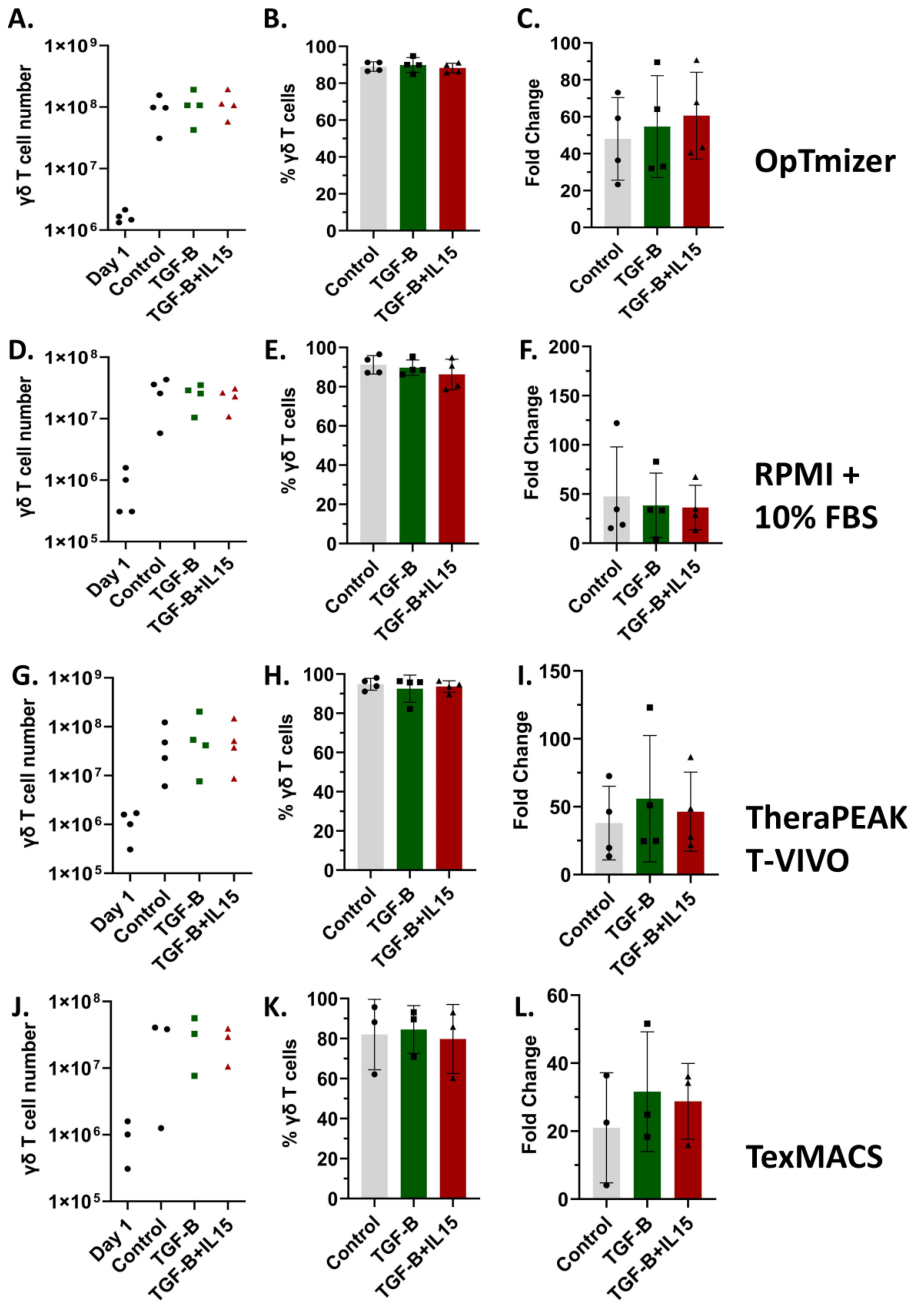
Vγ9Vδ2 γδ T cell expansions across multiple media formulations. Healthy donor PBMCs were activated with zoledronate and then cultured for 12 days in SFM containing IL-2 alone, IL-2 + TGF-β, or IL-2 + TGF-β + IL-15. Percentage of γδ T cells was measured in PBMCs (day 1) and after expansion in indicated cytokines. 15 expansions representing 11 unique donors were characterized. All expansions were performed in triplicate. Optimizer medium used **(A-C)** Error Bars represent SD. n = 4 experimental replicates. RPMI medium used **(D-F)** Error Bars represent SD. n = 4 experimental replicates. Lonza T-Vivo medium used **(G-I)** Error Bars represent SD. n = 4 experimental replicates. TexMACS medium used **(J-L)** Error Bars represent SD. n = 3 experimental replicates. **(A, D, G, J)** Number of γδ T cells on Day 12 of the expansion under Control, TGF-β, or TGF-β + IL-15 conditions. **(B, E, H, K)** Percent of γδ T cells following expansion under Control, TGF-β, or TGF-β + IL-15 conditions, showing high purity across all groups. **(C, F, I, L)** Fold change in cell number post expansion relative to starting cell count for each condition.

In addition to combination regimens using agents like ZOL and chemotherapy that may sensitize OS to γδ T cell recognition and killing, directing their phenotype from terminal effectors to a more memory-like phenotype has been shown to provide benefits for *in vivo* applications (22, 34). Therefore, the characterization of γδ T cells, particularly their memory phenotypes, is crucial for understanding their potential efficacy and persistence in cancer immunotherapy (54). The role of TGF-β is especially significant in shaping γδ T cell function. While TGF-β is known for its immunosuppressive effects within the tumor microenvironment, its influence on γδ T cells is multifaceted, as it affects their differentiation, homing capacity, and anti-tumor activity (22, 34, 55). To characterize the phenotypic effects of TGF-β and IL-15 on γδ T cell expansion, we assessed the proliferation, receptors, and differentiation status of cells cultured under our standard control (ZOL+ IL-2), TGF-β (ZOL + IL-2 + TGF-β) and TGF-β + IL-15 (ZOL + IL-2 + TGF-β+ IL-15) conditions, in various serum free conditions (OpTmizer serum-free media (SFM), TheraPEAK T-VIVO, and TexMACS) and RPMI + 10% FBS. Notably, TGF-β expanded cultures in OpTmizer achieved the highest absolute γδ T cell numbers at Day 12 with cell yields approaching 1 × 10^8^ to 1 × 10^9^ cells (**Figure 2A**). All conditions resulted in high γδ T cell purity, with minimal variation between groups, reaching ~90% in most cases (**Figures 2B, E, H, K**). TGF-β, although potentially immunosuppressive, does not impair Vδ2 γδ T cell expansion *in vitro* (**Figures 2C, F, I, L**). Cultures in RPMI + 10% FBS showed more variability, with lower overall cell numbers and reduced fold change, despite the high γδ T cell purity (**Figures 2D-F**). Under our conditions, serum free TexMACS supported the lowest expansion (**Figures 2J-L**).

**Figure 3:**
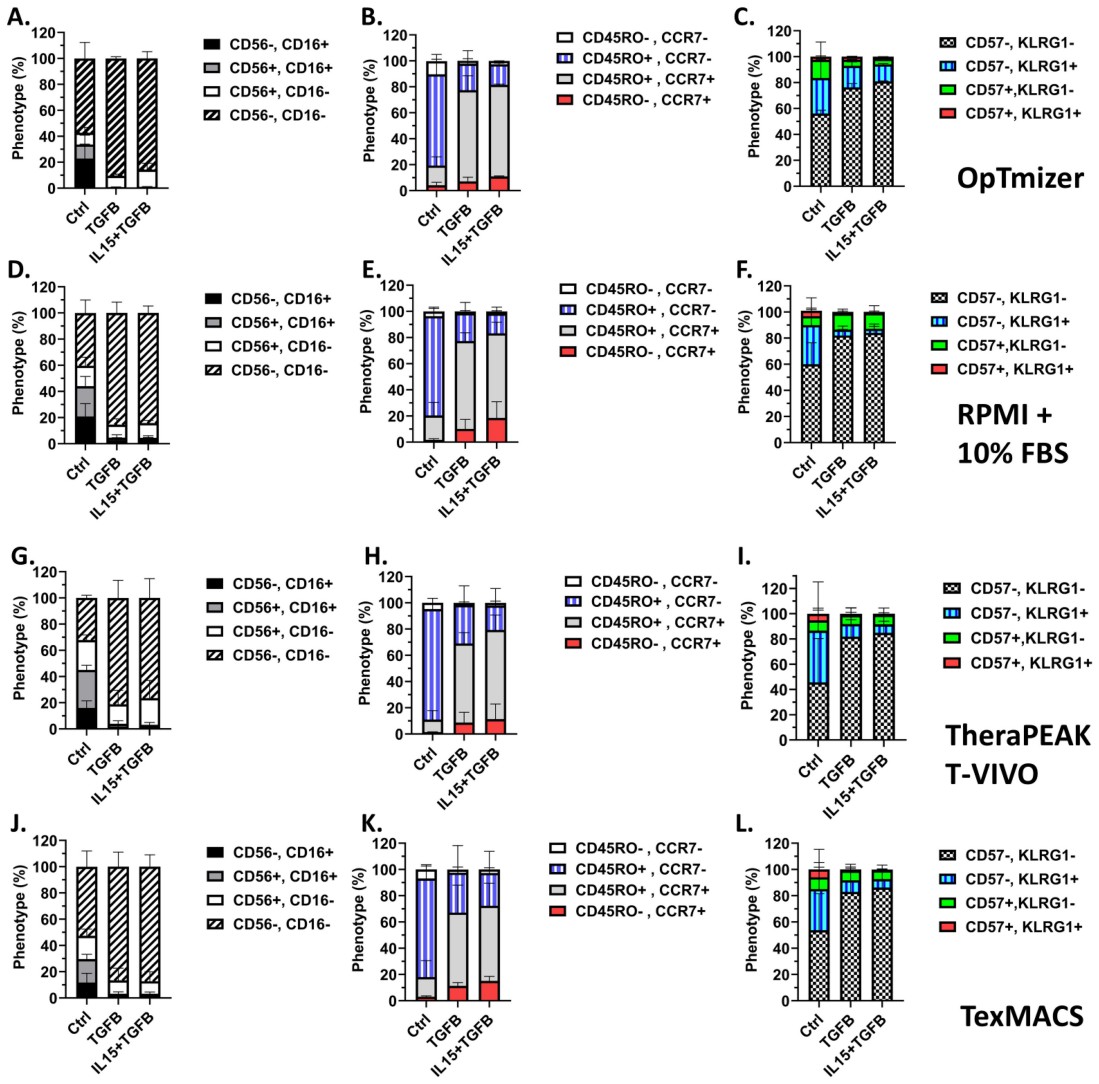
TGF-β modulates a multitude of markers on Vγ9Vδ2 γδ T cells in a media-independent manner. 15 expansions representing 11 unique donors were characterized. All expansions were performed in triplicate. Optimizer medium used **(A-C)** Error Bars represent SD. n= 4 experimental replicates. RPMI medium used **(D-F)** Error Bars represent SD. n = 4 experimental replicates. Lonza T-Vivo medium used **(G-I)** Error Bars represent SD. n = 4 experimental replicates. TexMACS medium used **(J-L)** Error Bars represent SD. n = 3 experimental replicates. **(A, D, G, J)** Expression of CD56 and CD16 on Day 12 γδ T cells following each expansion condition. **(B, E, H, K)** Memory phenotypes were assessed by CD45RO and CCR7 expression on the cell surface via flow cytometric analysis for each condition. **(C, F, I, L)** Terminal differentiation and activation were assessed by KLRG1 and CD57 expression on the cell surface via flow cytometric analysis for each condition. A two-way ANOVA with Tukey-Kramer *post hoc* analysis was done on these data. Tukey’s statistical analysis can be found in the Supplemental Stats File.

Phenotypic analysis showed that across all media tested, γδ T cells expanded in TGF-β or TGF-β + IL-15 conditions demonstrated a predominantly CD56-CD16- profile (**Figures 3A, D, G, J**). This suggests a consistent suppression of CD56 and CD16 expression by TGF-β irrespective of media. Memory phenotyping based on CD45RO and CCR7 expression also demonstrated a substantial shift in response to the cytokine condition. Under control conditions, γδ T cells predominantly displayed an effector memory phenotype (CD45RO+CCR7-), while there was a significant shift toward central memory (CD45RO+CCR7+) in the TGF-β and TGF-β + IL-15 expanded γδ T cells (**Figures 3B, E, H, K**). Interestingly, there is a slight, but non-significant, increase in the naïve-like phenotype (CD4RO-CCR7+) across the media types, except in the RPMI + 10% FBS medium where it is significant. This highlights the influence of TGF-β in driving phenotypic change (**Figures 3B, E, H, K**). Additionally, analysis of terminal differentiation and late-stage activation markers, KLRG1 and CD57, revealed the majority of γδ T cells across all conditions are CD57-KLRG1-, however, the TGF-β and TGF-β + IL-15 expanded γδ T cells are significantly enriched for this CD57-KLRG1- phenotype, indicating minimal senescence or exhaustion (**Figures 3C, F, I, L**). Interestingly, even though flow cytometry confirmed a marked decrease in NKG2D expression in the TGF-β expanded γδ T cells compared to the control expanded γδ T cells, these cells retained comparable cytolytic activity to control expanded γδ T cells across all treatment concentrations of Pal-IFO and E:T ratios (**Supplementary Figures 3E-H**). These results suggest that while NKG2D is a known activating receptor for γδ T cell-mediated killing, its downregulation does not abrogate cytotoxicity under these expansion conditions. No significant trends were observed across all media types when monitoring memory markers CD62L and CD27 when gated on CD45RO+ γδ T cells (**Supplementary Figures 4A-D**). However, when gated on this population of CD45RO+ γδ T cells and viewing CD62L and CD27 expression, the yellow and red color on the heatmap via FlowJo shows more CCR7 expression in the TGF-β and TGF-β + IL-15 expanded γδ T cells compared to the majority green on the control expanded γδ T cells which shows less CCR7 expression (**Supplementary Figure 4E**).

Despite these marked alterations in memory and surface marker phenotypes, standard expanded and TGF-β expanded γδ T cells retained robust cytotoxic function against both 143B and U2OS osteosarcoma cell lines (**Supplementary Figures 5A, B**). At both E:T ratios tested (1:1 and 5:1), The cytotoxicity of the TGF-β expanded γδ T cells was not significantly different when compared to control γδ T cells. Together, these results demonstrate TGF-β and TGF-β + IL-15 expanded γδ T cells have a more central memory-enriched phenotype and retain robust cytolytic potential.

The memory phenotype of the γδ T cells, particularly the CD45RO+ CCR7+ population, exhibited significant changes following a 4-hour cytotoxicity assay (**Supplementary Figure 6A**). Specifically, while both Control and TGF-β expanded γδ T cells demonstrated a reduction in their central memory-like phenotype post-killing, the TGF-β expanded γδ T cells retained significantly higher frequencies of this central memory-like phenotype compared to control γδ T cells (**Supplementary Figure 6A**). Overall, the expression patterns of CD62L, CD27, CD56, CD16, CD57, and KLRG1 after the cytotoxic assay mostly mirrored the pre-assay phenotypes within each group (**Supplementary Figures 6B, D**). This indicates the cytotoxic potential in γδ T cells may be uncoupled from the canonical memory surface marker profiles and highlights the resilience and functional versatility of γδ T cells.

### TGF-β expanded γδ T cells exhibit superior *In Vivo* persistence before and after cryopreservation

**Figure 4:**
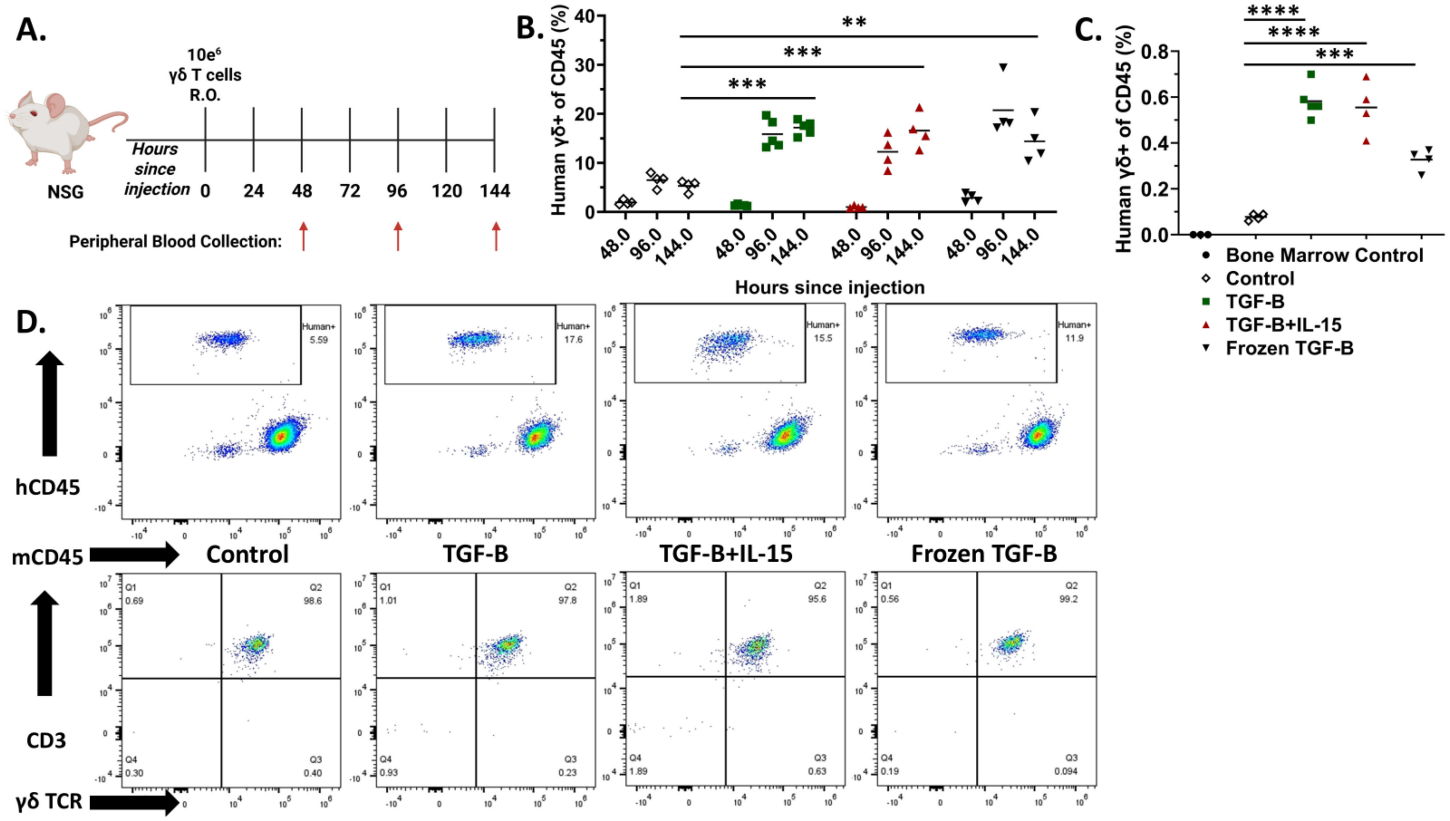
TGF-β expanded Vγ9Vδ2 γδ T cells have increased persistence compared to their standard counterparts. **(A)** Schematic of the experimental timeline and blood collection. NSG mice were injected intravenously via retro-orbital route with 1 × 107 γδ T cells under control conditions, with TGF-β, TGF-β + IL-15, or with TGF-β and then frozen and thawed. Peripheral blood was collected at 48-, 96-, and 144-hours post-injection (n = 4 - 5, n = 1 donor). **(B)** Individual percentages of CD3+ γδ TCR+ cells (gated on hCD45+, mCD45-) within the peripheral blood at each timepoint. White Diamond = Control expanded γδ T cells, Green Square = TGF-β expanded γδ T cells, Red Triangle = TGF-β + IL-15 expanded γδ T cells, and Black Upside-Down Triangle = Frozen TGF-β expanded γδ T cells. Statistical analysis represents A one-way ANOVA with Tukey's post hoc analysis was done on these data. (** p < 0.01, *** p < 0.001). Tukey's statistical analysis can be found in the Supplemental Stats File. **(C)** Percentages of CD3+ γδTCR+ cells (gated on hCD45+, mCD45-) detected in the bone marrow at 144 hours post-injection. Black Circle = Mouse Bone Marrow Control cells, White Diamond = Control expanded γδ T cells, Green Square = TGF-β expanded γδ T cells, Red Triangle = TGF-β + IL-15 expanded γδ T cells, and Black Upside-Down Triangle = Frozen TGF-β expanded γδ T cells. Statistical analysis represents A one-way ANOVA with Tukey's post hoc analysis was done on these data. (** p < 0.01, *** p < 0.001, **** p < 0.0001). Tukey's statistical analysis can be found in the Supplemental Stats File. **(D)** Representative flow cytometry plots of hCD45 vs mCD45 and CD3 vs γδ TCR expression in peripheral blood at 144 hours, showing CD3+γδTCR+ cell populations in each group.

Given the changes in memory phenotypes induced by TGF-β expanded γδ T cells, we next determined the kinetics of their *in vivo* persistence and engraftment potential. NSG mice were administered (i.v.) 1 × 10^7^ γδ T cells expanded under control conditions, TGF-β, TGF-β combined with IL-15, or TGF-β expanded cells that were frozen and subsequently thawed. Peripheral blood was collected every two days for six days to monitor circulating γδ T cells (**Figure 4A**). Flow cytometric analysis showed γδ T cells expanded in the presence of TGF-β displayed significantly higher frequencies in peripheral blood at both 96 and 144 hours compared to control-expanded γδ T cells (**Figure 4B**). Notably, while TGF-β and TGF-β + IL-15 expanded γδ T cells showed increasing trends in their percentages and fold changes over time, frozen then thawed TGF-β expanded γδ T cells peaked at 96 hours and slightly declined by 144 hours, suggesting a partial reduction in proliferative capacity or survival post-thaw (**Supplementary Figures 7A, B**). Despite this, all TGF-β expanded groups, including the frozen cohort, demonstrated markedly enhanced infiltration into the bone marrow at Day 6, compared to control-expanded γδ T cells (**Figure 4C**). Representative flow cytometry plots confirmed robust CD3+ γδTCR+ cell populations in the peripheral blood of all groups on Day 6 (**Figure 4D**). Collectively, these findings indicate TGF-β expanded γδ T cells exhibit increased *in vivo* persistence and bone marrow infiltration, with retained functionality after cryopreservation, supporting their potential as effective, off-the-shelf cellular immunotherapies.

### TGF-β expanded γδ T cells have enhanced anti-tumor efficacy in a metastatic osteosarcoma lung model

**Figure 5:**
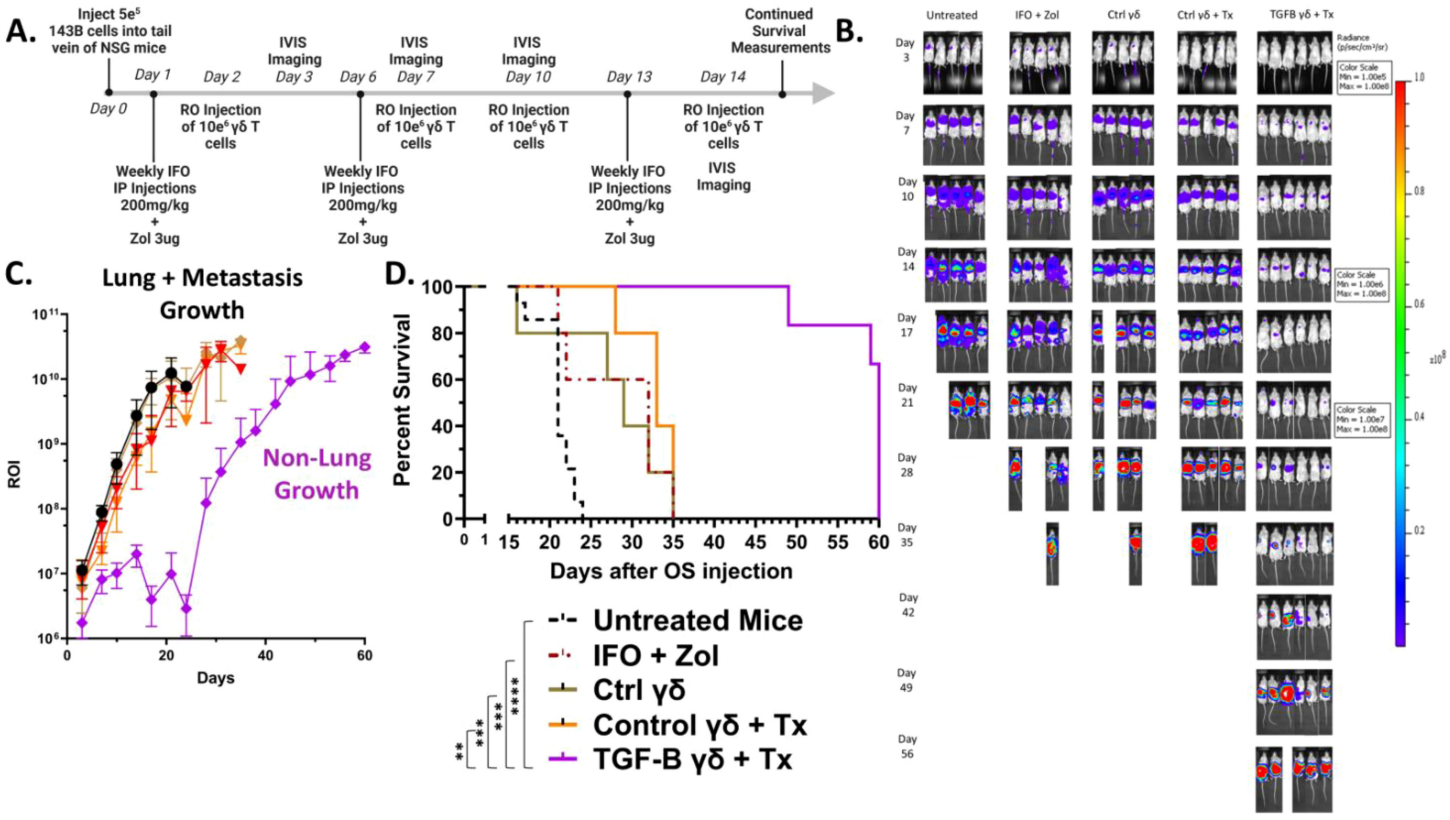
TGF-β expanded Vγ9Vδ2 γδ T cells combined with IFO and ZOL treatment suppress osteosarcoma progression and significantly improve survival *in vivo*. (Individual plots of Flux from Luciferase models per group showing 5–6 replicates) **(A)** Schematic. 5×10^5^ luciferase-expressing 143B osteosarcoma cells were injected via IV on Day 0. Day 1 mice were treated IP with IFO (200mg/kg) and ZOL (3ug) or PBS. Day 2, mice were dosed with 1x10^7^ γδ T cells via RO. Mice were subsequently treated with IFO and ZOL weekly, and γδ T cells twice a week for a total of 3 IFO and ZOL treatments, and 4 γδ T cell injections. Control γδ T cells alone were prepared and expanded the same as Control γδ T cells + Treatment (Tx). Treatment (Tx) is the addition of IFO (200mg/kg) and ZOL (3ug) to the therapy regiment. Serial IVIS ROI emissions from mice are shown. n = 14 Untreated, n = 5 IFO + ZOL, n =5 γδ T cells alone, n = 5 Control γδ T cells + Treatment (Tx), n = 6 TGF-β expanded γδ T cells + Treatment (Tx). **(B)** Representative serial IVIS ROI emissions from mice are shown. IVIS images of mice using three different scales throughout the experiment are presented: Days 3-10 are 1e5-1e8, Days 14-17 are 1e6-1e8, Days 21-56 are 1e7-1e8. Experimental groups shown: Untreated, IFO + ZOL, γδ T cells alone, Control γδ T cells + Treatment (Tx), TGF-β expanded γδ T cells + Treatment (Tx). **(C)** Mean IVIS ROI emission from each group is plotted overtime. Untreated = Black, IFO + ZOL = Red, γδ T cells alone = Brown, Control γδ T cells + Treatment (Tx) = Orange, TGF-β expanded γδ T cells + Treatment (Tx) = Purple. Error bars represent SD. **(D)** Kaplan-Meier survival analysis of data shown in **(A–D)** using a log-rank [Mantel-Cox] test. (** p < 0.01, *** p < 0.001, **** p < 0.0001).

To evaluate the *in vivo* anti-tumor efficacy of γδ T cells against an aggressive metastatic OS model, NSG mice were intravenously injected via the tail vein with 5×10^5^ luciferase-expressing 143B osteosarcoma cells on Day 0. Beginning on Day 1, mice were treated intraperitoneally with 200mg/kg IFO and 3μg ZOL, or PBS as a control. On Day 2, 1×10^7^ γδ T cells were administered intravenously via retro-orbital route. Mice continued to receive weekly doses of IFO and ZOL, along with twice weekly infusions of γδ T cells, totaling three chemotherapy treatments and four γδ T cell doses. IVIS bioluminescent imaging was performed twice weekly to monitor tumor progression (**Figure 5A**). Mouse weight remained relatively constant for all groups until the week of their deaths, where it started to decline (**Supplementary Figure 8A**). Interestingly, IVIS bioluminescent imaging revealed the TGF-β combined treatment group showed marked suppression of tumor growth in the lungs with residual or relapsing tumor burden being predominantly observed in the liver rather than pulmonary tissue (**Supplementary Figure 8B**). Among all groups, mice treated with TGF-β expanded γδ T cells with IFO and Zol (TGF-β combined treatment) exhibited pronounced delay in tumor development, as shown by the reduced bioluminescent signal intensity (**Figures 5B,C** and **Supplementary Figures 8C-G**). Although all treated groups showed some survival advantage over untreated mice, only the TGF-β combined treatment group exhibited both significant tumor suppression and increased survival, with mice living past 50 days (**Figure 5D**). This shift in tumor localization was evident in IVIS images, where signal intensity for tumors treated with TGF-β expanded cells was no longer concentrated in the thoracic region but instead emerged within the abdominal area, consistent with hepatic involvement. We hypothesize this improved tumor control is driven by the enhanced persistence of the TGF-β expanded γδ T cells, as supported by peripheral blood analysis demonstrating higher γδ T cell percentages in the TGF-β group four days after initial infusion (**Supplementary Figures 9A, B**). To inform potential risks of this therapeutic strategy, a safety assessment was completed by a certified veterinarian on three mice one week after they had been administered two doses of 1×10^7^ TGF-β expanded γδ T cells. No significant findings were found in the organs of these animals, except for the lungs which showed signs consistent with CO_2_ asphyxiation (**Supplementary Figure 10**). These findings highlight the *in vivo* anti-tumor activity of the TGF-β expanded γδ T cells to clear the primary site of metastatic seeding and underscore the therapeutic relevance of memory phenotype and persistence in the treatment of OS lung metastases using adoptive γδ T cell therapy.

Therefore, TGF-β expanded γδ T cells, when combined with the tumor-sensitizing agents ZOL and IFO, exhibit potent anti-tumor effects in a metastatic OS lung model, leading to reduce tumor burden and significantly improved survival, which supports the therapeutic potential of TGF-β driven γδ T cell expansion strategies for adoptive cell therapy in relapse/refractory OS.

## Discussion

We show CI using Vγ9Vδ2 γδ T cells represent a promising avenue for the treatment of OS, particularly in the context of lung metastasis, where standard therapies fall short (6, 9, 56–58). Our findings demonstrate *ex vivo* expansion of γδ T cells in the presence of TGF-β, with or without IL-15, reprograms these cells towards a memory-enriched phenotype characterized by increased expression of memory markers, decreased expression of senescence and activation markers, prolonged persistence *in vivo*, and enhanced infiltration into the bone marrow. Despite reduced expression of canonical cytotoxic markers CD16 and CD56, TGF-β expanded γδ T cells displayed superior anti-tumor efficacy compared to standard IL2/ZOL expanded γδ T cells in an OS lung metastasis model when combined with ZOL and IFO. This therapeutic combination led to improved tumor control in the lungs and significantly increased survival, highlighting the importance of persistence, memory, and a decrease in senescent markers in γδ T cell-based immunotherapies.

Mechanistically, ZOL preconditioning of OS cells enhanced their susceptibility to γδ T cell mediated lysis, likely through the accumulation of phosphoantigens such as IPP, which are recognized via the butyrophilin 3A1 γδ TCR axis (59, 60). While ZOL did not significantly upregulate NKG2D ligands or decrease OS cell viability directly, its ability to condition tumor cells for γδ TCR-dependent killing underscores the importance of antigen-specific recognition in mediating therapeutic efficacy.

Pal-IFO did increase NKG2D ligand expression in a dose-dependent manner. The necessity for adding chemotherapy to the therapeutic regimen came from preliminary *in vivo* studies using a subcutaneous OS model being treated with γδ T cells combined with ZOL, which showed capacity to control tumor growth and modestly improve survival. However, the overall therapeutic effect remained limited. Tumor volumes continued to increase over time, and survival benefits, although statistically significant compared to the untreated group, did not indicate robust or durable tumor regression. These findings suggest γδ T cell-based immunotherapy, even when augmented by ZOL, may not be sufficient as a standalone treatment in an aggressive OS model. The results are consistent with our previous neuroblastoma (NB) results where we show γδ T cells alone do not control tumor growth, but only when the tumor is conditioned with chemotherapy is the γδ T cell-based CI effective. For our ongoing NCT05400603 clinical trial, proper conditions to sensitize the NB tumor, modulate the tumor microenvironment, and optimize adoptive γδ T cell responses are being tested.

Interestingly, CD16 is downregulated in TGF-β expanded cells, so antibody-based therapies relying on CD16 expression to activate antibody-dependent cellular cytotoxicity (ADCC) may not be an ideal combination. However, γδ T cells do bridge innate and adaptive immunity, so the addition of antibody-based therapies should not be overlooked. The current data along with our clinical trial may inform future design of tailored strategies that improve persistence, efficacy, and safety of γδ T cell immunotherapies across diverse solid tumors.

Previous studies have greatly contributed to the γδ T cell-based immunotherapy field against solid tumors (22, 29, 31, 34, 35, 61, 62). Our study focuses on how to enhance γδ T cell-based chemoimmunotherapy regimens, using TGF-β in *in vitro* expansions, which induced a pronounced shift toward a memory-enriched phenotype, as evidenced by an increased population of CD45RO+ CCR7+ (central memory) compared to standard IL2/ZOL expanded γδ T cells. This phenotypic transition was accompanied by modest changes in terminal differentiation markers, with most of the cells retaining a CD57-KLRG1- profile. This is indicative of a less exhausted and more proliferative population. Importantly, co-administration of IL-15 further enhanced the fold expansion without compromising the central memory phenotype. These findings suggest TGF-β creates a more durable and responsive γδ T cell product suitable for adoptive immunotherapy. To evaluate the persistence of these cells *in vivo*, we administered NSG mice with γδ T cells expanded under our various conditions. TGF-β expanded γδ T cells, both fresh and cryopreserved, exhibited greater persistence in peripheral blood and enhanced infiltration into the bone marrow compared to control γδ T cells. This persistence may be related to the memory phenotype and could have contributed to their enhanced anti-tumor function.

In the context of tumor challenge, TGF-β expanded γδ T cells exerted superior control over metastatic OS progression. In a lung metastasis model using luciferase-tagged 143B OS cells, mice treated with TGF-β γδ T cells in combination with ZOL and IFO exhibited significant tumor growth delay and improved overall survival. While all treatment groups receiving either ZOL and IFO and/or γδ T cells showed improved survival over the untreated control mice, only the TGF-β combined treatment group demonstrated a sustained reduction in tumor burden and a statistically significant survival benefit compared to all other groups. Based on *in vitro* findings, this effect was potentially driven in part by the γδ TCR-dependent recognition due to the sensitization of ZOL, the upregulated NKG2D ligands due to IFO, and the increased persistence of the TGF-β γδ T cells.

Although we did not assess γδ T cell infiltration in the lungs or liver in this study, prior reports suggest γδ T cells preferentially traffic to the lungs in response to inflammation or infection and can comprise a substantial portion of resident pulmonary lymphocytes (63–66). In contrast, the liver is predominantly enriched with Vδ1+ γδ T cells, and Vγ9Vδ2 γδ T cells are notably depleted in hepatic malignancies (67, 68).

Our findings suggest TGF-β drives a selective modulation of memory-associated markers in Vγ9Vδ2 γδ T cells, notably upregulating CCR7 without concurrent increases in CD62L or CD27. This phenotype likely represents a transitional or noncanonical memory-like state, which may not align with classical definition of central memory (69). Importantly, the TGF-B expanded γδ T cells exhibit reduced expression of senescence markers (CD57, KLRG1), suggesting improved proliferative potential and persistence. Prior studies have shown CCR7 expression itself can promote both immune surveillance and signaling pathways essential for T cell function (70, 71). Thus, this transitional profile may reflect a functionally advantageous state, apart from traditional memory classification, that supports enhanced *in vivo* persistence, and tumor clearance potential.

While CCR7 upregulation in TGF-β expanded γδ T cells may suggest altered trafficking toward secondary lymphoid tissues, we propose this transitional phenotype supports persistence and activation potential without necessarily impeding tumor infiltration, especially in models lacking structed lymphoid organs. Future studies using humanized or orthotopic models will help clarify the role of CCR7 in γδ T cell localization and function *in vivo*, particular in the context of CAR-redirected γδ T cells.

Interestingly, γδ T cells expanded in the presence of TGF-β exhibited a significant decrease in the surface expression of CD16 and CD56, two markers traditionally associated with cytotoxic effector functions and antibody-dependent cellular cytotoxicity(ADCC) (72, 73). In the TGF-β expanded cultures, CD56+CD16+ populations were nearly absent. The reduction in classic cytotoxic markers did not impair, and actually enhanced, the overall *in vivo* efficacy of the TGF-β expanded γδ T cells. Rather than relying on immediate, innate effector functions such as ADCC, these T cells may function through sustained, antigen-dependent cytotoxicity. This mechanism could be supported by the increased persistence, central memory phenotype, and TCR-dependent killing observed in the ZOL-sensitized OS cytotoxicity *in vitro* assays. These findings challenge the traditional assumption that high CD16/CD56 expression is required for γδ T cell potency (74–76), highlighting that durable therapeutic efficacy may instead stem from enhanced TCR-driven responses and longevity within the tumor-bearing host.

While our data show TGF-β induces a favorable memory phenotype in γδ T cells with enhanced *in vivo* persistence and anti-tumor activity, as mentioned above, this does come at the cost of combining this therapy with a monoclonal antibody, due to the lack of CD16 expression. To address this, future studies should explore strategies that modulate TGF-β signaling in a context dependent manner. For instance, TGF-β agonists could be employed during the γδ T cell expansion instead of TGF-β to induce memory differentiation without fully repressing CD16 or CD56 expression (77). Alternatively, short-term priming with TGF-β, followed by a cytokine rescue phase using IL-15 and/or IL-21, might preserve memory programing while recovering effector functions. This concept is supported by prior studies showing transient TGF-β exposure is sufficient to induce stem-like memory characteristics in CD8+ T cells (78), and modulation of downstream signaling pathways such as SMAD2/3 can separate exhaustion from memory induction in αβ T cells (79, 80). Another potential avenue, given the clinical relevance of ADCC in combination therapies using monoclonal antibodies (31, 81), would be to engineer γδ T cells to restore CD16 expression post-expansion through a CD16-CAR, either via mRNA electroporation or lentiviral transduction (82, 83).

Recent studies have established similar importance for cytokine-modulation of γδ T cell function and persistence with enhanced anti-tumor potential. Mo et al., demonstrated culturing CAR-engineered Vδ2 T cells in human platelet lysate, which contains TGF-β (84), supplemented media reduces cellular senescence and apoptosis, ultimately boosting *in vivo* anti-tumor activity against B-cell acute lymphoblastic leukemia (B-ALL) (34). Beatson et al., showed *ex vivo* exposure of γδ T cells to TGF-β induces reprogramming into a less differentiated, central memory-like phenotype with superior proliferative capacity, cytokine production, and cytotoxicity against leukemic, ovarian, and breast cancer cell lines (22). Both studies underscore the capacity of TGF-β expanded γδ T cell’s anti-tumor efficacy in liquid tumors or general immunogenic cancers, and we now show similar results using an immune-cold OS cancer. Fowler et al., engineered Vγ9Vδ2 γδ T cells to co-secrete GD2-specific scFv-Fc opsonin and a synthetic IL-15 fusion protein, creating an “armed” γδ T-cell platform that showed superior persistence and tumor control compared to unmodified γδ T cells in an orthotopic patient-derived osteosarcoma model (29). Notably, this was achieved through a single injection of 1 × 10^7^ 14G2a stIL15- OPS- γδ T cells plus ZOL 7 days later. With over 80% event-free survival for this cellular immunotherapy, this study highlights the importance of combining direct cytolysis with bystander activation in a solid tumor setting. Interestingly, they used RPMI with 10% FBS for their expansions, which may have contributed to the high CD16 expression on their γδ T cells, while our study focused on the use of OpTmizer, a serum free medium, allowing for it to be a clinically relevant product candidate.

Building on foundational principles and understanding of Vγ9Vδ2 γδ T cells, the present study extends the therapeutic scope of TGF-β expanded cells by applying them to OS, a poorly immunogenic malignancy with high relapse rates, deadly metastatic locations, and limited immunotherapeutic options. We demonstrate TGF-β expanded γδ T cells exhibit a stable central memory-like phenotype, a decrease in the terminal effector-like phenotype, and high γδ T cell purity across multiple GMP-compliant, serum-free media platforms. These findings help support, and reinforce, the functional benefits of TGF-β conditioning in expansion conditions. Furthermore, this work uniquely integrates γδ T cell programming within a chemoimmunotherapy framework that includes ZOL and IFO, agents that sensitize OS to γδ T cells. Together, these results help translate the memory-enhancing benefits of TGF-β signaling into a solid tumor setting and support clinical strategies targeting metastatic pediatric solid tumors.

## Data Availability

The original contributions presented in the study are included in the article/**Supplementary Material**. Further inquiries can be directed to the corresponding author/s.

## Supplementary material

Supplementary Figure 1 Schematic of the Mevalonate pathway and mRNA expression levels of BTN2A1 and BTN3A1 in Osteosarcoma. (A) Schematic of the Mevalonate pathway in an osteosarcoma tumor cell after treatment with Zoledronic Acid causing the upregulation of Isopentenyl Pyrophosphate, which induces a confirmation change in the butyrophilin complex, allowing for recognition by the Vδ2 γδ TCR. (B-C) mRNA expression levels of BTN2A1 and BTN3A1 in osteosarcoma tissues compared to normal tissues, based on publicly available datasets obtained from R2: Genomics Analysis and Visualization Platform (https://r2.amc.nl). One way ANOVA (* p < 0.05). Error bars represent SD.

Supplementary Figure 2 Vγ9Vδ2 γδ T cells increase survival in NSG mice in a subcutaneous *in vivo* flank Osteosarcoma model. **(A)** Schematic. 5x10^6 143B cells were injected into the flank, subcutaneously, of NSG mice on Day 0. Over 10 days the tumor volume would grow to reach between 100-300mm^3. Once the volume reached over 100mm^3 the mice on ~Day 10 would receive 1x10^7 γδ T cells via direct injection into the tumor site. Mice were subsequently treated with γδ T cells twice a week for a total of 4 γδ T cell injections. Injections either contained 1x10^7 γδ T cells + ZOL (3ug) or 1x10^7 γδ T cells alone. Tumor burden and weight was monitored multiple times a week. n = 5 Untreated, n = 3 γδ T cells, n = 3 γδ T cells + ZOL. Error bars represent SD. **(B)** Mean tumor growth for each group is plotted overtime. Error bars represent SD. **(C)** Kaplan-Meier survival analysis of data shown in B using a log-rank [Mantel-Cox] test.

Supplementary Figure 3 TGF-β reduces NKG2D expression on Vγ9Vδ2 γδ T cells without impairing their cytotoxicity against Pal-IFO conditioned osteosarcoma cells’ elevated NKG2D ligand expression. **(A-B)** mRNA expression levels of NKG2D ligands (ULBP1 and MICA/MICB) in osteosarcoma tissues compared to normal tissues, based on publicly available datasets obtained from R2: Genomics Analysis and Visualization Platform (https://r2.amc.nl). One way ANOVA (** p < 0.01). Error bars represent SD. **(C-D)** Quantification of stress antigen surface expression in 143B and U2OS cells following Pal-IFO treatment, represented as mean fluorescence intensity (MFI). ≥50% cell death was observed at 1000 µM. Cell death measured via eFluor780. Error bars represent SD. n = 2 experimental replicates. **(E-F)** 143B were conditioned overnight with Pal-IFO (0-500 µM). After treatment with Pal-IFO or control, osteosarcoma cells were incubated with day 12 Control or TGF-β expanded Vγ9Vδ2 γδ T cells for 4h at 1:1 **(E)** and 5:1 **(F)** effector to target ratios (E:T). Viability was measured via eFluor780 and Annexin V. Error bars represent SD. n = 3 technical replicates. **(G)** Flow cytometry plots showing % of NKD2G+ cells in Control and TGF-β expanded Vγ9Vδ2 γδ T cells. **(H)** Flow cytometry histogram displaying NKG2D expression on the surface of Control and TGF-β expanded Vγ9Vδ2 γδ T cells. Blue = Control expanded γδ T cells and Red = TGF-β γδ T cells. Student’s T-test (** p < 0.01).

Supplementary Figure 4 Variety among traditional memory markers in various media types in Vγ9Vδ2 γδ T cells. **(A-D)** Expression of CD27 and CD62L gated on CD45RO+ γδ T cells for the various media types. **(A)** OpTmizer n = 4 experimental replicates, **(B)** RPMI + FBS 10% n = 4 experimental replicates, **(C)** TheraPEAK T-Vivo n = 4 experimental replicates, **(D)** TexMACS n = 3 experimental replicates. Error bars represent SD. **(E**) Representative flow cytometry plots showing expression of CD27 and CD62L, with a CCR7 heatmap expression, gated on CD45RO+ γδ T cells expanded in OpTmizer. A two-way ANOVA with Tukey-Kramer *post hoc* analysis was done on this experiment. Tukey’s statistical analysis can be found in the Supplemental Stats File.

Supplementary Figure 5 Control and TGF-β expanded Vγ9Vδ2 γδ T cells exhibit similar cytotoxicity against osteosarcoma. Osteosarcoma cell lines (**(A)**143B and **(B)** U2OS) were co-cultured with frozen then thawed day 12 *ex vivo* expanded γδ T cells for 4h at 1:1 and 5:1 E:T ratios. After 4h viability was measured via flow cytometry (Annexin-V and eFluor780). Error bars represent SD. n = 5–8 experimental replicates. n = 3 donors.

Supplementary Figure 6 Phenotype characteristics of Vγ9Vδ2 γδ T cells after a 4-hour cytotoxicity assay. Osteosarcoma cells, 143B, were co-cultured with frozen then thawed day 12 *ex vivo* expanded γδ T cells for 4h at 1:1 and 5:1 E:T ratios. After 4h, Standard expanded γδ T cells (IL2/ZOL) and TGF-β expanded γδ T cells (IL2/ZOL/TGF-β) were analyzed by flow cytometry for various phenotypic markers: **(A)** CD45RO and CCR7, **(B)** CD27 and CD62L, **(C)** CD57 and KLRG1, and **(D)** CD56 and CD16. n = 1 biological and n = 3 technical. A two-way ANOVA with Tukey-Kramer *post hoc* analysis was done on this experiment. Tukey’s statistical analysis can be found in the Supplemental Stats File.

Supplementary Figure 7 Various expansion strategies of Vγ9Vδ2 γδ T cells persistence in NSG mice. **(A)** Kinetics of γδ+ cell percentages in peripheral blood over time. White Diamond = Control, Green Square = TGF-β, Red Triangle = TGF-β + IL-15, and Black Upside-Down Triangle = Frozen TGF-β. Error bars represent SD. n = 4 technical replicates for all groups. **(B)** Fold change in γδ+ cells starting at the various timepoints. **(C)** Flow cytometry gating strategy for finding γδ+ cell populations in peripheral blood. **(D)** Representative flow cytometry plots of hCD45 vs mCD45 and then CD3 vs γδ TCR expression when gated upon hCD45+ in peripheral blood at 48 hours. **(E)** Representative flow cytometry plots of hCD45 vs mCD45 and then CD3 vs γδ TCR expression when gated upon hCD45+ in peripheral blood at 96 hours.

Supplementary Figure 8 TGF-β expanded Vγ9Vδ2 γδ T cells have decreased tumor burden compared to their standard counterparts. **(A)** Mean IVIS ROI emission from each group is plotted overtime. Error bars represent SD. **(B)** Day 7 Untreated mouse compared to and overlaid against a Day 35 TGF-β γδ Treated mouse. **(C-G)** Individual serial IVIS ROI emissions from mice are shown for each mouse per group. Error bars represent SD.

Supplementary Figure 9 TGF-β expanded Vγ9Vδ2 γδ T cells have increased persistence in tumor bearing mice 96 hours after treatment. **(A)** Representative flow cytometry plots of hCD45 vs mCD45 and then CD3 vs γδ TCR expression when gated upon hCD45+ in peripheral blood at 96 hours post initial γδ T cell injection. **(B)** Individual percentages of CD3+ γδ TCR+ cells (gated on hCD45+, mCD45-) within the peripheral blood for each group. (n = 3). Black Triangle = γδ T cells Alone, Grey Circle = Control γδ T cells + Treatment, Green Square = TGF-β expanded γδ T cells + Treatment.

Supplementary Figure 10 Individual animal gross necropsy report after TGF-β expanded γδ T cell therapy.

Supplementary Figure 11 Statistical analysis of the data shown in **Figures 1**, **3**, **4**, **5**, and **Supplementary Figures 2**, **4**, and **6**.
