# Supplementary material for: Central memory-enriched Vγ9Vδ2 γδ T cells via TGF-β expansion demonstrate enhanced in vivo efficacy against metastatic osteosarcoma
Source: Front Immunol. 2025 Sep 3;16:1657760. doi: 10.3389/fimmu.2025.1657760 (PMC12440941; doi:10.3389/fimmu.2025.1657760)
Supplement: Supplementary Figure 1 — Schematic of the Mevalonate pathway and mRNA expression levels of BTN2A1 and BTN3A1 in Osteosarcoma. (A) Schematic of the Mevalonate pathway in an osteosarcoma tumor cell after treatment with Zoledronic Acid causing the upregulation of Isopentenyl Pyrophosphate, which induces a confirmation change in the butyrophilin complex, allowing for recognition by the Vδ2 γδ TCR. (B-C) mRNA expression levels of BTN2A1 and BTN3A1 in osteosarcoma tissues compared to normal tissues, based on publicly available datasets obtained from R2: Genomics Analysis and Visualization Platform (https://r2.amc.nl). One way ANOVA (* p < 0.05). Error bars represent SD. [file DataSheet1.pdf]

Supplemental Table 1: All flow-cytometry antibodies used in this study. Antibodies denoted with an asterisk were used in the γδ phenotype expansion experiments.

Antibody	Clone	Fluorophore	Vendor	Catalog #
CD3*	UCHT1	BV421	BD Biosciences	562426
CD62L*	DREG-56	BV510	BioLegend	304844
CD45RO*	UCHL1	BV650	BioLegend	304231
CD27*	M-T271	BV711	BD Biosciences	564893
KLRG-1*	13F12F2	BV786	ThermoFisher	417-9488-42
CCR7*	G043H7	FITC	BioLegend	353216
CD45RA*	HI100	PerCP-Cy5.5	BD Biosciences	563429
γδTCR*	B1	PE	BioLegend	331210
CD57*	HNK-1	Alexa Fluor 594	BioLegend	359626
CD16*	3G8	PE-Cyanine7	BioLegend	302016
CD56*	5.1H11	APC	BioLegend	362503
eFluor780*		APC-eFluor 780	ThermoFisher	65-0865-18
Annexin V		APC	BioLegend	640941
Violet Proliferation Dye 450		V450	BD Biosciences	562158
MICA/B	6D4	PE	BioLegend	320906
ULBP-1		Alexa Fluor 488	R&D Systems	FAB1380G
ULBP-2/5/6		APC	R&D Systems	FAB1298A
TRAIL-R1	S35-934	PE	BD Biosciences	564180
TRAIL-R2		Alexa Fluor 488	R&D Systems	FAB6311G
Mouse IgG2a, κ Isotype Ctrl Antibody	MOPC-173	PE	BioLegend	400212
Mouse IgG2a, κ Isotype Ctrl Antibody	MOPC-173	Alexa Fluor 488	BioLegend	400233
Mouse IgG2a, κ Isotype Ctrl Antibody	MOPC-173	APC	BioLegend	400220
hCD45	HI30	FITC	BD Biosciences	555482
mCD45	30-F11	BV510	BioLegend	103138

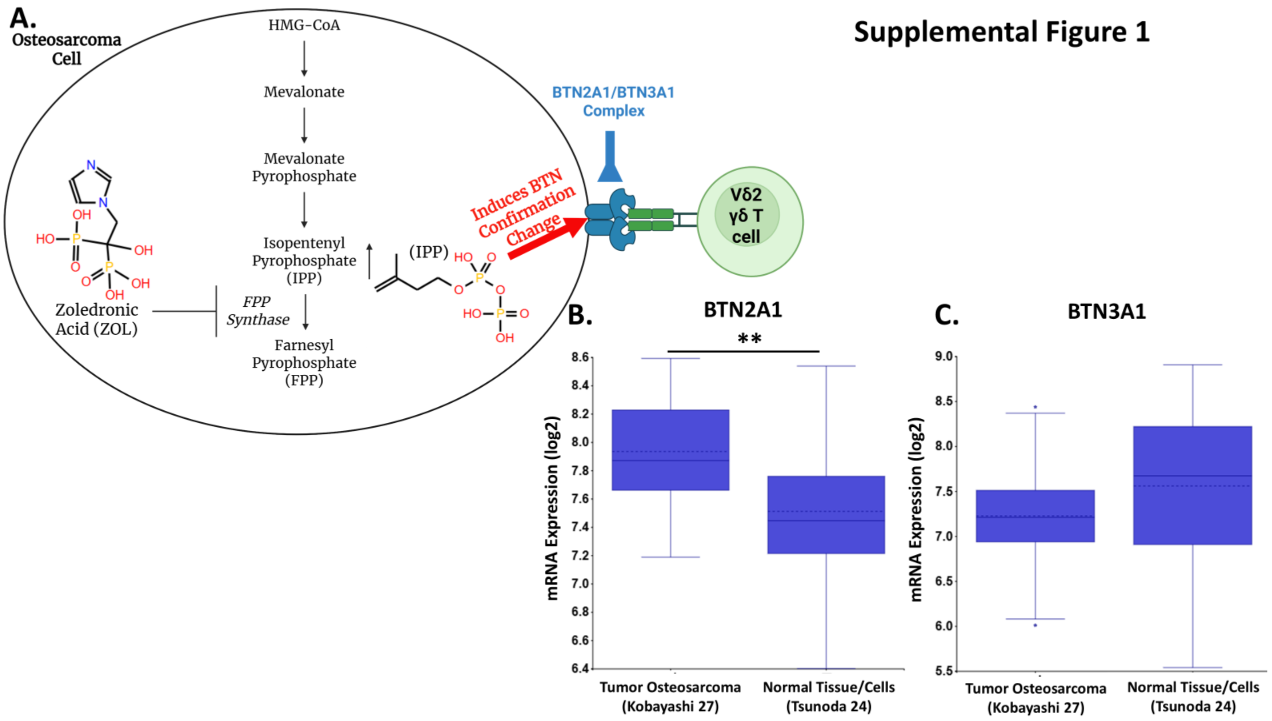

Supplemental Figure 1. Schematic of the Mevalonate pathway and mRNA expression levels of BTN2A1 and BTN3A1 in Osteosarcoma. (A) Schematic of the Mevalonate pathway in an osteosarcoma tumor cell after treatment with Zoledronic Acid causing the upregulation of Isopentenyl Pyrophosphate, which induces a confirmation change in the butyrophilin complex, allowing for recognition by the Vδ2 γδ TCR. **(B-C)** mRNA expression levels of BTN2A1 and BTN3A1 in osteosarcoma tissues compared to normal tissues, based on publicly available datasets obtained from R2: Genomics Analysis and Visualization Platform (https://r2.amc.nl). One way ANOVA (* $p < 0.05$). Error bars represent SD.

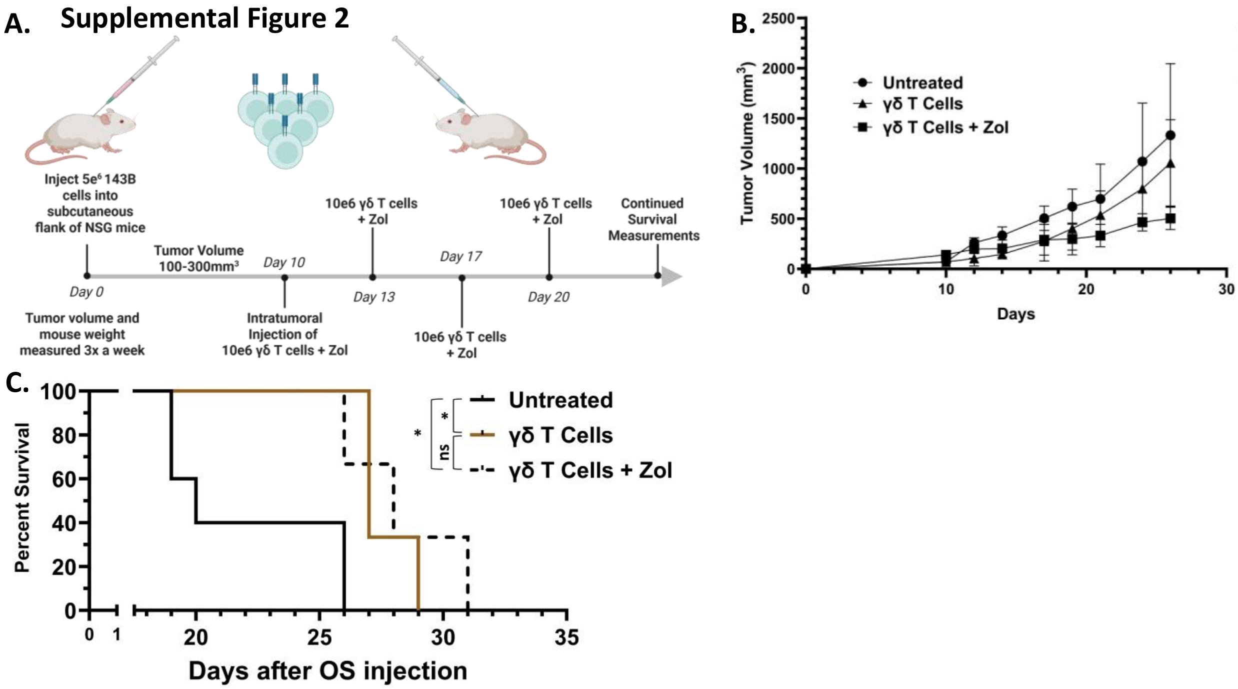

Supplemental Figure 2. Vγ9Vδ2 γδ T cells increase survival in NSG mice in a subcutaneous *in vivo* flank Osteosarcoma model. (A) Schematic. 5x10^6 143B cells were injected into the flank, subcutaneously, of NSG mice on Day 0. Over 10 days the tumor volume would grow to reach between 100-300mm^3. Once the volume reached over 100mm^3 the mice on ~Day 10 would receive 1x10^7 γδ T cells via direct injection into the tumor site. Mice were subsequently treated with γδ T cells twice a week for a total of 4 γδ T cell injections. Injections either contained 1x10^7 γδ T cells + ZOL (3ug) or 1x10^7 γδ T cells alone. Tumor burden and weight was monitored multiple times a week. n = 5 Untreated, n = 3 γδ T cells, n = 3 γδ T cells + ZOL. Error bars represent SD. **(B)** Mean tumor growth for each group is plotted overtime. Error bars represent SD. **(C)** Kaplan-Meier survival analysis of data shown in B using a log-rank [Mantel-Cox] test.

Supplemental Figure 3

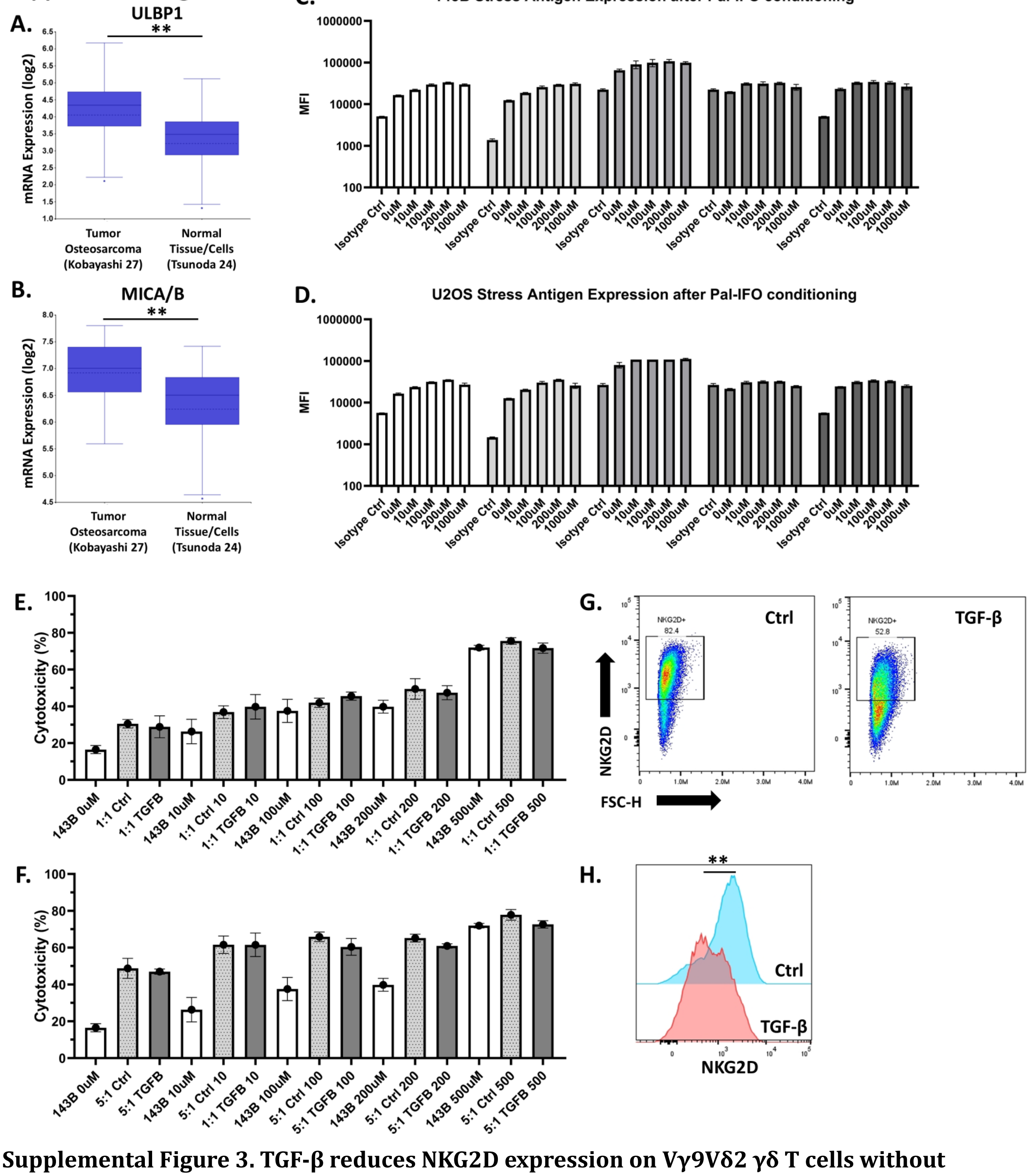

Supplemental Figure 3. TGF-β reduces NKG2D expression on Vγ9Vδ2 γδ T cells without impairing their cytotoxicity against Pal-IFO conditioned osteosarcoma cells' elevated NKG2D ligand expression. **(A-B)** mRNA expression levels of NKG2D ligands (ULBP1 and MICA/MICB) in osteosarcoma tissues compared to normal tissues, based on publicly available datasets obtained from R2: Genomics Analysis and Visualization Platform (https://r2.amc.nl). One way ANOVA (** $p < 0.01$). Error bars represent SD. **(C-D)** Quantification of stress antigen surface expression in 143B

and U2OS cells following Pal-IFO treatment, represented as mean fluorescence intensity (MFI). ≥50% cell death was observed at 1000 μM. Cell death measured via eFluor780. Error bars represent SD. n = 2 experimental replicates. **(E-F) 143B** were conditioned overnight with Pal-IFO (0-500 μM). After treatment with Pal-IFO or control, osteosarcoma cells were incubated with day 12 Control or TGF-β expanded Vγ9Vδ2 γδ T cells for 4h at 1:1 **(E)** and 5:1 **(F)** effector to target ratios (E:T). Viability was measured via eFluor780 and Annexin V. Error bars represent SD. n = 3 technical replicates. **(G)** Flow cytometry plots showing % of NKG2D+ cells in Control and TGF-β expanded Vγ9Vδ2 γδ T cells. **(H)** Flow cytometry histogram displaying NKG2D expression on the surface of Control and TGF-β expanded Vγ9Vδ2 γδ T cells. Blue = Control expanded γδ T cells and Red = TGF-β γδ T cells. Student's T-test (** $p < 0.01$). n = 3 technical replicates.

Supplemental Figure 4

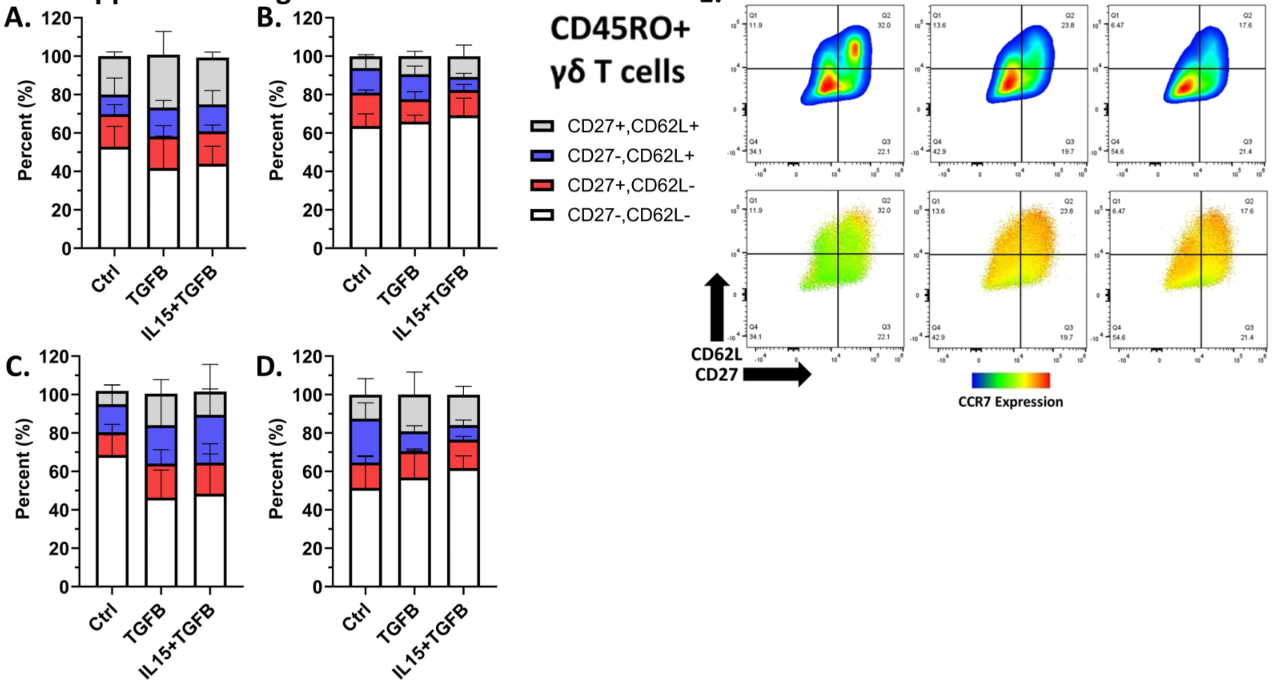

Supplemental Figure 4. Variety among traditional memory markers in various media types in Vγ9Vδ2 γδ T cells. (A-D) Expression of CD27 and CD62L gated on CD45RO+ γδ T cells for the various media types. **(A)** OpTmizer n = 4 experimental replicates, **(B)** RPMI + FBS 10% n = 4 experimental replicates, **(C)** TheraPEAK T-Vivo n = 4 experimental replicates, **(D)** TexMACS n = 3 experimental replicates. Error bars represent SD. **(E)** Representative flow cytometry plots showing expression of CD27 and CD62L, with a CCR7 heatmap expression, gated on CD45RO+ γδ T cells expanded in OpTmizer. A two-way ANOVA with Tukey-Kramer post hoc analysis was done on this experiment. Tukey's statistical analysis can be found in the Supplemental Stats File.

Supplemental Figure 5

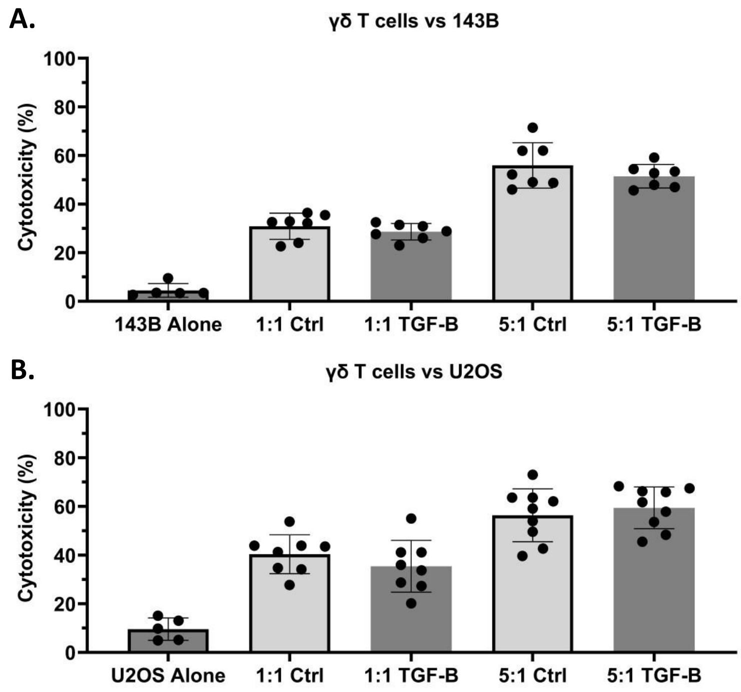

Supplemental Figure 5. Control and TGF-β expanded Vγ9Vδ2 γδ T cells exhibit similar cytotoxicity against osteosarcoma. Osteosarcoma cell lines (**(A)143B** and **(B) U2OS**) were co-cultured with frozen then thawed day 12 *ex vivo* expanded γδ T cells for 4h at 1:1 and 5:1 E:T ratios. After 4h viability was measured via flow cytometry (Annexin-V and eFluor780). Error bars represent SD. n = 5 – 8 experimental replicates. n = 3 donors.

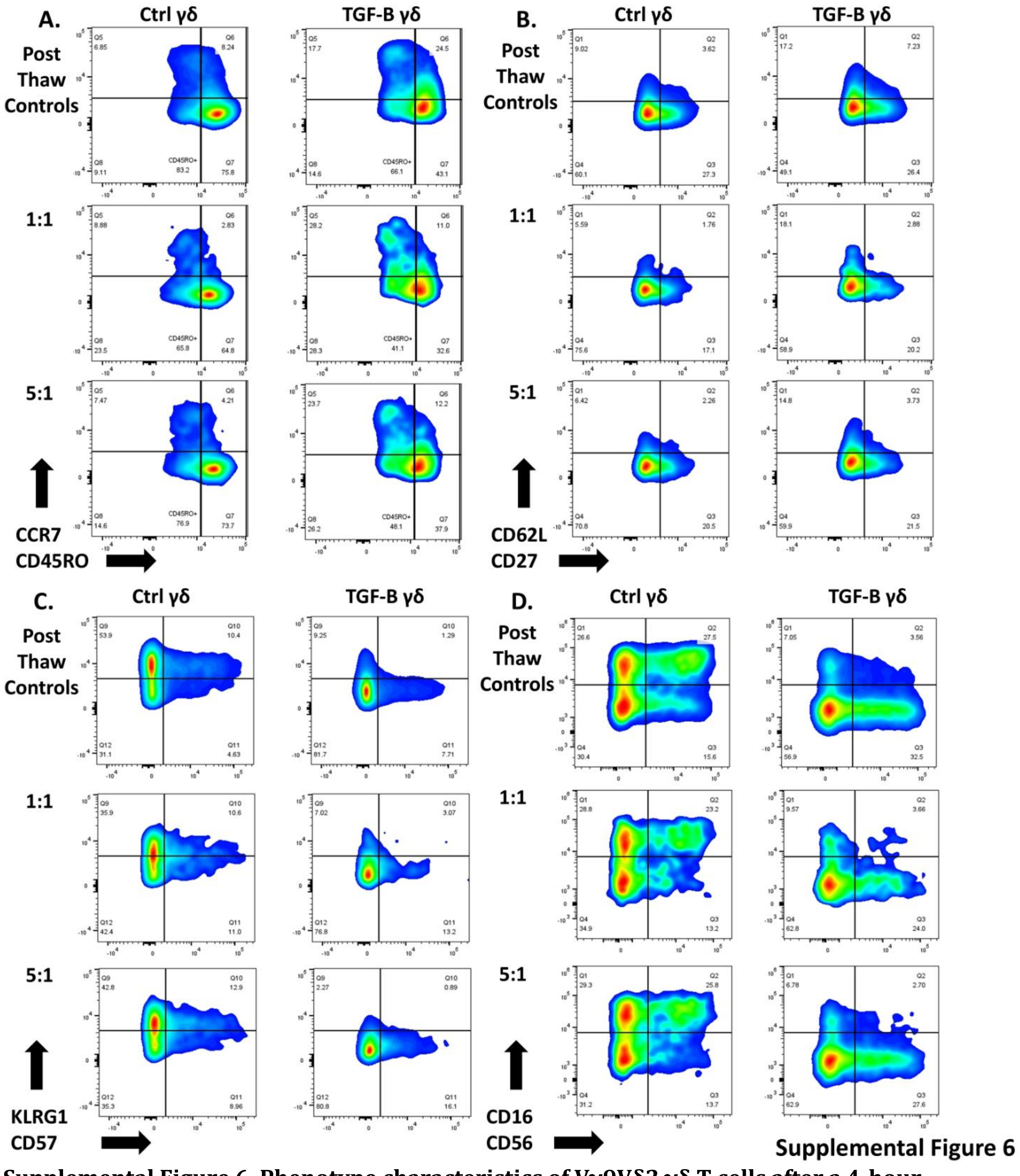

Supplemental Figure 6. Phenotype characteristics of Vγ9Vδ2 γδ T cells after a 4-hour cytotoxicity assay. Osteosarcoma cells, 143B, were co-cultured with frozen then thawed day 12 *ex vivo* expanded γδ T cells for 4h at 1:1 and 5:1 E:T ratios. After 4h, Standard expanded γδ T cells (IL2/ZOL) and TGF-β expanded γδ T cells (IL2/ZOL/TGF-β) were analyzed by flow cytometry for various phenotypic markers: **(A)** CD45RO and CCR7, **(B)** CD27 and CD62L, **(C)** CD57 and KLRG1, and **(D)** CD56 and CD16. n = 1 biological and n = 3 technical. A two-way ANOVA with Tukey-Kramer post hoc analysis was done on this experiment. Tukey's statistical analysis can be found in the Supplemental Stats File.

Supplemental Figure 7

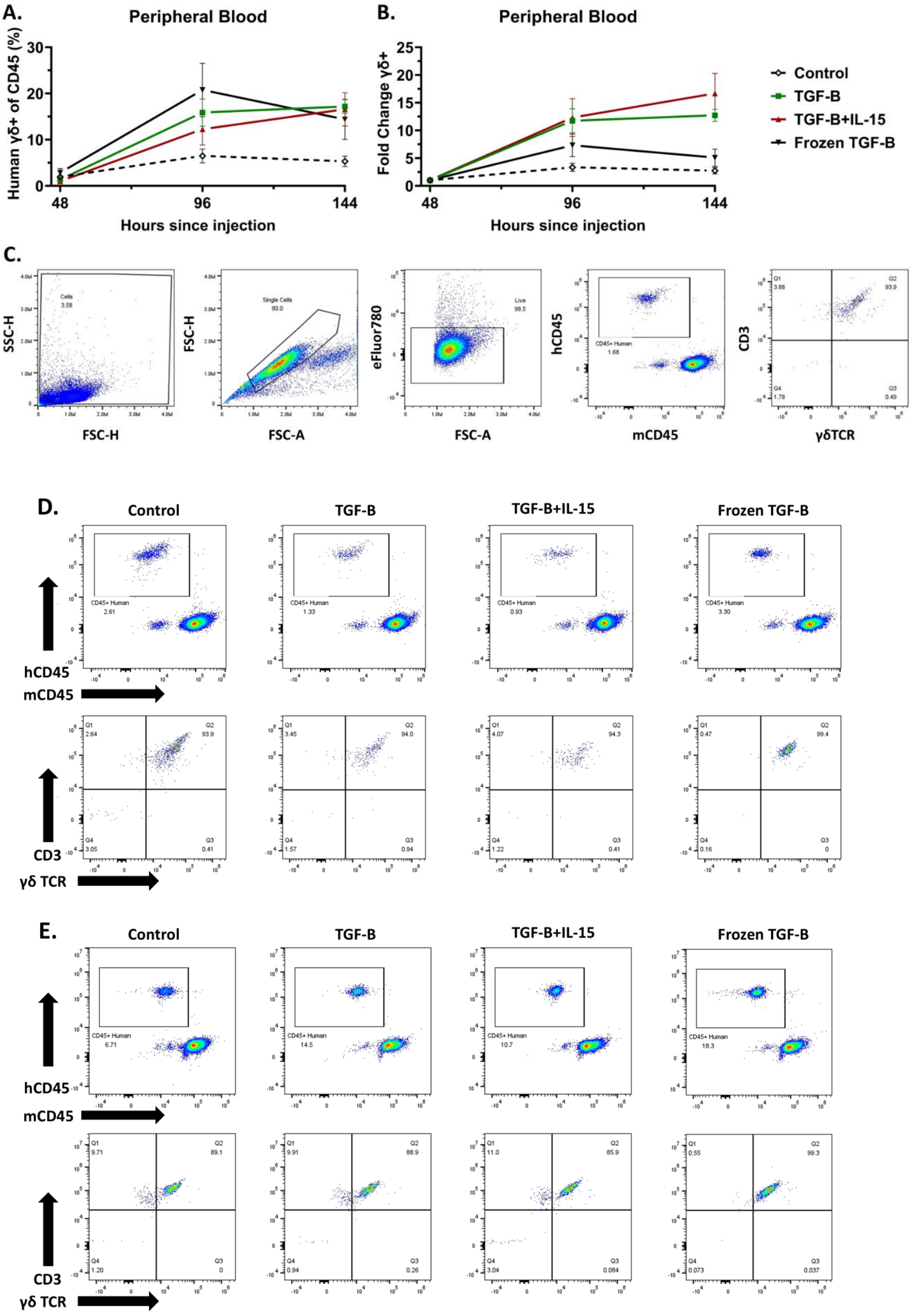

Supplemental Figure 7. Various expansion strategies of Vγ9Vδ2 γδ T cells persistence in NSG mice. (A) Kinetics of γδ+ cell percentages in peripheral blood over time. White Diamond = Control, Green Square = TGF-β, Red Triangle = TGF-β + IL-15, and Black Upside-Down Triangle = Frozen TGF-β. Error bars represent SD. n = 4 technical replicates for all groups. **(B)** Fold change in γδ+ cells starting at the various timepoints. **(C)** Flow cytometry gating strategy for finding γδ+ cell populations in peripheral blood. **(D)** Representative flow cytometry plots of hCD45 vs mCD45 and then CD3 vs γδ TCR expression when gated upon hCD45+ in peripheral blood at 48 hours. **(E)** Representative flow cytometry plots of hCD45 vs mCD45 and then CD3 vs γδ TCR expression when gated upon hCD45+ in peripheral blood at 96 hours.

Supplemental Figure 8

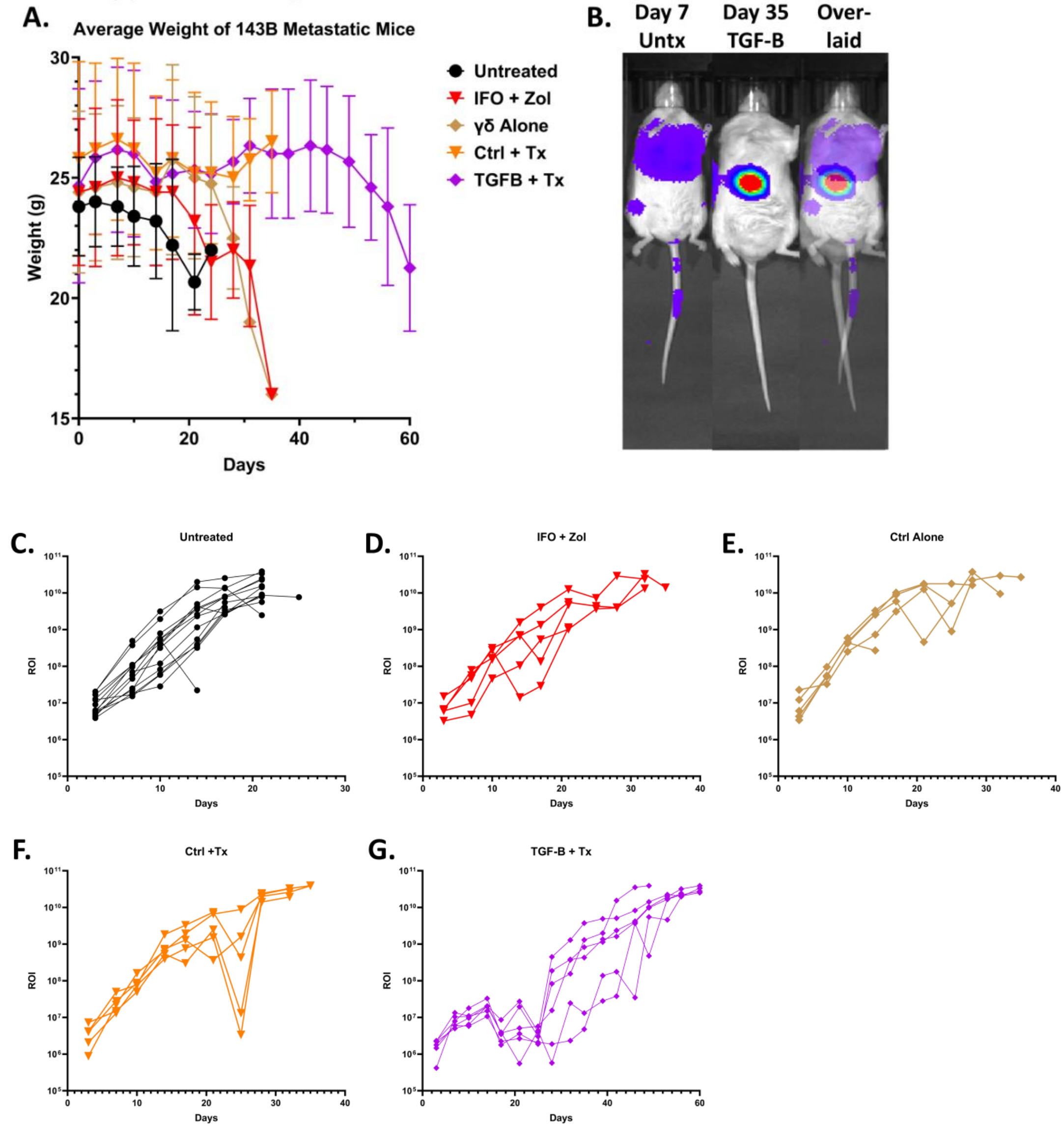

Supplemental Figure 8. TGF-β expanded Vγ9Vδ2 γδ T cells have decreased tumor burden compared to their standard counterparts. (A) Mean IVIS ROI emission from each group is plotted overtime. Error bars represent SD. **(B)** Day 7 Untreated mouse compared to and overlaid against a Day 35 TGF-β γδ Treated mouse. **(C-G)** Individual serial IVIS ROI emissions from mice are shown for each mouse per group. Error bars represent SD.

Supplemental Figure 9

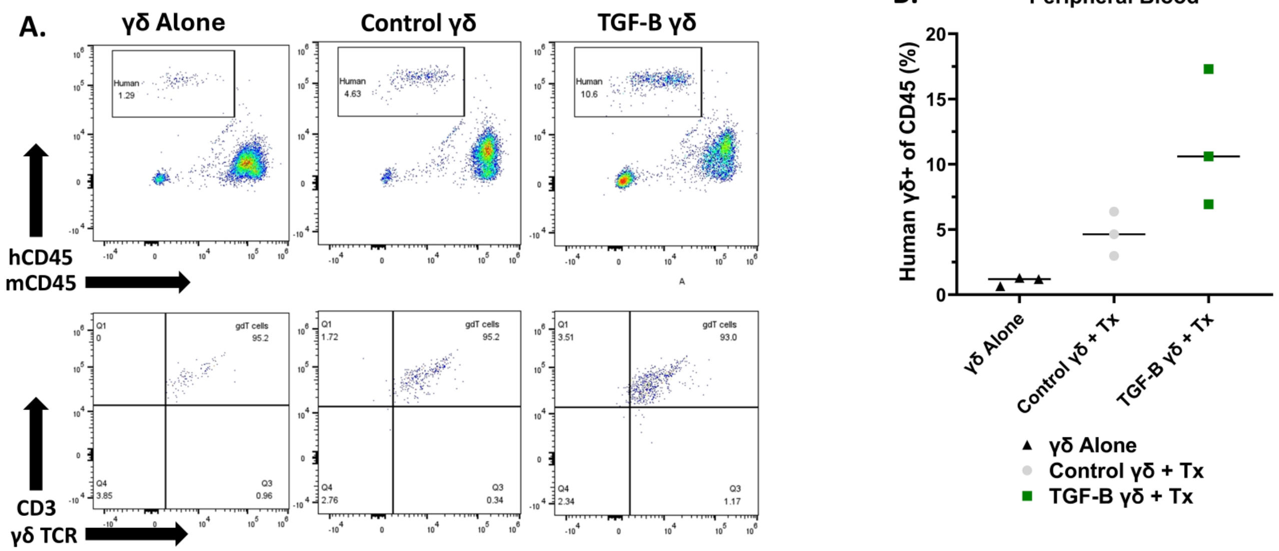

Supplemental Figure 9. TGF-β expanded Vγ9Vδ2 γδ T cells have increased persistence in tumor bearing mice 96 hours after treatment. (A) Representative flow cytometry plots of hCD45 vs mCD45 and then CD3 vs γδ TCR expression when gated upon hCD45+ in peripheral blood at 96 hours post initial γδ T cell injection. **(B)** Individual percentages of CD3+ γδ TCR+ cells (gated on hCD45+, mCD45-) within the peripheral blood for each group. (n = 3). Black Triangle = γδ T cells Alone, Grey Circle = Control γδ T cells + Treatment, Green Square = TGF-β expanded γδ T cells + Treatment.

Supplemental Figure 10. Individual animal gross necropsy report after TGF-β expanded γδ T cell therapy.

Cage Card	Weight	Tissue	Strain	DOB	Sex	Comments
3571679	27 grams	Liver	NSG	06/05/2025	Male	No significant findings
3571679	27 grams	Spleen	NSG	06/05/2025	Male	No significant findings
3571679	27 grams	Kidneys	NSG	06/05/2025	Male	No significant findings
3571679	27 grams	Lungs	NSG	06/05/2025	Male	Multifocal mottled dark red discoloration consistent with means of euthanasia (CO_2 asphyxiation)
3571679	27 grams	Heart	NSG	06/05/2025	Male	No significant findings
3571679	27 grams	Intestines	NSG	06/05/2025	Male	No significant findings
3571679	27 grams	Stomach	NSG	06/05/2025	Male	No significant findings
3594112	23 grams	Liver	NSG	06/05/2025	Male	No significant findings
3594112	23 grams	Spleen	NSG	06/05/2025	Male	No significant findings
3594112	23 grams	Kidneys	NSG	06/05/2025	Male	No significant findings
3594112	23 grams	Lungs	NSG	06/05/2025	Male	Multifocal mottled dark red discoloration consistent with means of euthanasia (CO_2 asphyxiation)
3594112	23 grams	Heart	NSG	06/05/2025	Male	No significant findings
3594112	23 grams	Intestines	NSG	06/05/2025	Male	No significant findings
3594112	23 grams	Stomach	NSG	06/05/2025	Male	No significant findings
3594112	27 grams	Liver	NSG	06/05/2025	Male	No significant findings
3594112	27 grams	Spleen	NSG	06/05/2025	Male	No significant findings
3594112	27 grams	Kidneys	NSG	06/05/2025	Male	No significant findings
3594112	27 grams	Lungs	NSG	06/05/2025	Male	Multifocal mottled dark red discoloration consistent with means of euthanasia (CO_2 asphyxiation)
3594112	27 grams	Heart	NSG	06/05/2025	Male	No significant findings
3594112	27 grams	Intestines	NSG	06/05/2025	Male	No significant findings
3594112	27 grams	Stomach	NSG	06/05/2025	Male	No significant findings

Supplemental Figure 11. Statistical analysis of the data shown in Figures 1, 3, 4, 5, and Supplemental Figures 2, 4, and 6.

Figure 1A

Tukey's multiple comparisons test	Mean Diff.	95.00% CI of diff.	Below threshold?	Summary	Adjusted P Value
0uM vs. 2uM	-5.92	-24.07 to 12.23	No	ns	0.9405
0uM vs. 5uM	-9.02	-27.17 to 9.134	No	ns	0.6758
0uM vs. 10uM	-17.16	-35.31 to 0.9938	No	ns	0.071
0uM vs. 20uM	-9.09	-27.24 to 9.064	No	ns	0.668
0uM vs. 50uM	-49.39	-67.54 to -31.24	Yes	****	<0.0001
0uM vs. 100uM	-45.22	-63.37 to -27.07	Yes	****	<0.0001
0uM vs. 200uM	-49.6	-67.75 to -31.45	Yes	****	<0.0001
2uM vs. 5uM	-3.1	-21.25 to 15.05	No	ns	0.9985
2uM vs. 10uM	-11.24	-29.39 to 6.914	No	ns	0.4302
2uM vs. 20uM	-3.17	-21.32 to 14.98	No	ns	0.9983
2uM vs. 50uM	-43.47	-61.62 to -25.32	Yes	****	<0.0001
2uM vs. 100uM	-39.3	-57.45 to -21.15	Yes	****	<0.0001
2uM vs. 200uM	-43.68	-61.83 to -25.53	Yes	****	<0.0001
5uM vs. 10uM	-8.14	-26.29 to 10.01	No	ns	0.7698
5uM vs. 20uM	-0.07	-18.22 to 18.08	No	ns	>0.9999
5uM vs. 50uM	-40.37	-58.52 to -22.22	Yes	****	<0.0001
5uM vs. 100uM	-36.2	-54.35 to -18.05	Yes	****	<0.0001
5uM vs. 200uM	-40.58	-58.73 to -22.43	Yes	****	<0.0001
10uM vs. 20uM	8.07	-10.08 to 26.22	No	ns	0.7769
10uM vs. 50uM	-32.23	-50.38 to -14.08	Yes	***	0.0003
10uM vs. 100uM	-28.06	-46.21 to -9.906	Yes	**	0.0013
10uM vs. 200uM	-32.44	-50.59 to -14.29	Yes	***	0.0003
20uM vs. 50uM	-40.3	-58.45 to -22.15	Yes	****	<0.0001
20uM vs. 100uM	-36.13	-54.28 to -17.98	Yes	****	<0.0001
20uM vs. 200uM	-40.51	-58.66 to -22.36	Yes	****	<0.0001
50uM vs. 100uM	4.17	-13.98 to 22.32	No	ns	0.991
50uM vs. 200uM	-0.21	-18.36 to 17.94	No	ns	>0.9999
100uM vs. 200uM	-4.38	-22.53 to 13.77	No	ns	0.988

Figure 1B

Tukey's multiple comparisons test	Mean Diff.	95.00% CI of diff.	Below threshold?	Summary	Adjusted P Value
0uM vs. 2uM	-9.98	-26.02 to 6.061	No	ns	0.4246
0uM vs. 5uM	-9.47	-25.51 to 6.571	No	ns	0.4855
0uM vs. 10uM	-7.33	-23.37 to 8.711	No	ns	0.7539
0uM vs. 20uM	-5.5	-21.54 to 10.54	No	ns	0.9244
0uM vs. 50uM	-23.79	-39.83 to -7.749	Yes	**	0.002
0uM vs. 100uM	-33.1	-49.14 to -17.06	Yes	****	<0.0001
0uM vs. 200uM	-37.94	-53.98 to -21.90	Yes	****	<0.0001
2uM vs. 5uM	0.51	-15.53 to 16.55	No	ns	>0.9999
2uM vs. 10uM	2.65	-13.39 to 18.69	No	ns	0.9988
2uM vs. 20uM	4.48	-11.56 to 20.52	No	ns	0.9731
2uM vs. 50uM	-13.81	-29.85 to 2.231	No	ns	0.1197
2uM vs. 100uM	-23.12	-39.16 to -7.079	Yes	**	0.0026
2uM vs. 200uM	-27.96	-44.00 to -11.92	Yes	***	0.0004
5uM vs. 10uM	2.14	-13.90 to 18.18	No	ns	0.9997
5uM vs. 20uM	3.97	-12.07 to 20.01	No	ns	0.9861
5uM vs. 50uM	-14.32	-30.36 to 1.721	No	ns	0.0986
5uM vs. 100uM	-23.63	-39.67 to -7.589	Yes	**	0.0021
5uM vs. 200uM	-28.47	-44.51 to -12.43	Yes	***	0.0003
10uM vs. 20uM	1.83	-14.21 to 17.87	No	ns	0.9999
10uM vs. 50uM	-16.46	-32.50 to -0.4191	Yes	*	0.0422
10uM vs. 100uM	-25.77	-41.81 to -9.729	Yes	***	0.0009
10uM vs. 200uM	-30.61	-46.65 to -14.57	Yes	***	0.0001
20uM vs. 50uM	-18.29	-34.33 to -2.249	Yes	*	0.0198
20uM vs. 100uM	-27.6	-43.64 to -11.56	Yes	***	0.0004
20uM vs. 200uM	-32.44	-48.48 to -16.40	Yes	****	<0.0001
50uM vs. 100uM	-9.31	-25.35 to 6.731	No	ns	0.5053
50uM vs. 200uM	-14.15	-30.19 to 1.891	No	ns	0.1052
100uM vs. 200uM	-4.84	-20.88 to 11.20	No	ns	0.9596

Figure 1C

Tukey's multiple comparisons test	Mean Diff.	95.00% CI of diff.	Below threshold?	Summary	Adjusted P Value
**1:1 γδ** vs. **1:1 γδ + Zol**	-23.79	-31.06 to -16.51	Yes	****	<0.0001
**1:1 γδ** vs. **5:1 γδ**	-15.21	-22.49 to -7.932	Yes	****	<0.0001
**1:1 γδ** vs. **5:1 γδ + Zol**	-48.43	-55.71 to -41.15	Yes	****	<0.0001
**1:1 γδ + Zol** vs. **5:1 γδ**	8.577	1.299 to 15.85	Yes	*	0.0153
**1:1 γδ + Zol** vs. **5:1 γδ + Zol**	-24.64	-31.92 to -17.36	Yes	****	<0.0001
**5:1 γδ** vs. **5:1 γδ + Zol**	-33.22	-40.50 to -25.94	Yes	****	<0.0001

Figure 1D

Tukey's multiple comparisons test	Mean Diff.	95.00% CI of diff.	Below threshold?	Summary	Adjusted P Value
**1:1 γδ** vs. **1:1 γδ + Zol**	-22.13	-27.42 to -16.84	Yes	****	<0.0001
**1:1 γδ** vs. **5:1 γδ**	-11.5	-16.79 to -6.211	Yes	****	<0.0001
**1:1 γδ** vs. **5:1 γδ + Zol**	-37.79	-43.07 to -32.50	Yes	****	<0.0001
**1:1 γδ + Zol** vs. **5:1 γδ**	10.63	5.344 to 15.92	Yes	****	<0.0001
**1:1 γδ + Zol** vs. **5:1 γδ + Zol**	-15.65	-20.94 to -10.36	Yes	****	<0.0001
**5:1 γδ** vs. **5:1 γδ + Zol**	-26.29	-31.57 to -21.00	Yes	****	<0.0001

Figure 3 Stats Tukey's multiple comparisons test	OpTmizer Predicted (LS) mean diff.	95.00% CI of diff	Below threshold?	Summary	Adjusted P Value
**CD56-, CD16+**					
Ctrl vs. TGFB	22.31	14.10 to 30.53	Yes	****	<0.0001
Ctrl vs. IL15+TGFB	22.26	14.04 to 30.47	Yes	****	<0.0001
TGFB vs. IL15+TGFB	-0.05833	-9.242 to 9.125	No	ns	0.9999
**CD56+, CD16+**					
Ctrl vs. TGFB	10.5	2.286 to 18.71	Yes	**	0.0086
Ctrl vs. IL15+TGFB	10.37	2.153 to 18.58	Yes	**	0.0096
TGFB vs. IL15+TGFB	-0.1333	-9.317 to 9.050	No	ns	0.9993
**CD56+, CD16-**					
Ctrl vs. TGFB	0.3053	-7.909 to 8.520	No	ns	0.9957
Ctrl vs. IL15+TGFB	-4.238	-12.45 to 3.976	No	ns	0.4374
TGFB vs. IL15+TGFB	-4.543	-13.73 to 4.640	No	ns	0.4671
**CD56-, CD16-**					
Ctrl vs. TGFB	-33.13	-41.34 to -24.91	Yes	****	<0.0001
Ctrl vs. IL15+TGFB	-28.39	-36.61 to -20.18	Yes	****	<0.0001
TGFB vs. IL15+TGFB	4.733	-4.450 to 13.92	No	ns	0.4381
**CD45RO- , CCR7+**					
Ctrl vs. TGFB	-2.998	-12.13 to 6.132	No	ns	0.7094
Ctrl vs. IL15+TGFB	-6.8	-17.01 to 3.408	No	ns	0.2517
TGFB vs. IL15+TGFB	-3.802	-14.01 to 6.406	No	ns	0.6437
**CD45RO+ , CCR7+**					
Ctrl vs. TGFB	-55.18	-64.31 to -46.04	Yes	****	<0.0001
Ctrl vs. IL15+TGFB	-55.42	-65.62 to -45.21	Yes	****	<0.0001
TGFB vs. IL15+TGFB	-0.2417	-10.45 to 9.966	No	ns	0.9982
**CD45RO+ , CCR7-**					
Ctrl vs. TGFB	49.9	40.77 to 59.03	Yes	****	<0.0001
Ctrl vs. IL15+TGFB	54.48	44.27 to 64.68	Yes	****	<0.0001
TGFB vs. IL15+TGFB	4.575	-5.633 to 14.78	No	ns	0.5298
**CD45RO- , CCR7-**					
Ctrl vs. TGFB	8.012	-1.119 to 17.14	No	ns	0.0962
Ctrl vs. IL15+TGFB	7.539	-2.669 to 17.75	No	ns	0.1857
TGFB vs. IL15+TGFB	-0.4725	-10.68 to 9.735	No	ns	0.9931

Figure 3 Stats<br>Tukey's multiple comparisons test	OpTmizer<br>Predicted (LS) mean diff.	95.00% CI of diff	Below threshold?	Summary	Adjusted P Value
**CD57-, KLRG1-**					
Ctrl vs. TGFB	-22.19	-33.33 to -11.05	Yes	****	<0.0001
Ctrl vs. IL15+TGFB	-22.66	-33.80 to -11.52	Yes	****	<0.0001
TGFB vs. IL15+TGFB	-0.475	-13.34 to 12.39	No	ns	0.9956
**CD57-, KLRG1+**					
Ctrl vs. TGFB	12.44	1.297 to 23.58	Yes	*	0.0253
Ctrl vs. IL15+TGFB	12.49	1.347 to 23.63	Yes	*	0.0246
TGFB vs. IL15+TGFB	0.05	-12.81 to 12.91	No	ns	>0.9999
**CD57+,KLRG1-**					
Ctrl vs. TGFB	9.365	-1.775 to 20.51	No	ns	0.1156
Ctrl vs. IL15+TGFB	9.628	-1.513 to 20.77	No	ns	0.1028
TGFB vs. IL15+TGFB	0.2625	-12.60 to 13.13	No	ns	0.9987
**CD57+, KLRG1+**					
Ctrl vs. TGFB	0.3663	-10.77 to 11.51	No	ns	0.9965
Ctrl vs. IL15+TGFB	0.5737	-10.57 to 11.71	No	ns	0.9915
TGFB vs. IL15+TGFB	0.2075	-12.66 to 13.07	No	ns	0.9992

OpTmizer	Mean	SD	Figure 3 Stats
**CD56-, CD16+**			
Ctrl	22.87	11.05	
TGFB	0.55	0.18	
IL-15+TGFB	0.61	0.24	
**CD56+, CD16+**			
Ctrl	10.82	7.12	
TGFB	0.32	0.10	
IL-15+TGFB	0.46	0.21	
**CD56+, CD16-**			
Ctrl	9.01	4.18	
TGFB	8.71	1.31	
IL-15+TGFB	13.25	4.93	
**CD56-, CD16-**			
Ctrl	57.29	12.35	
TGFB	90.42	1.39	
IL-15+TGFB	85.68	5.28	
**CD45RO- , CCR7+**			
Ctrl	4.15	2.33	
TGFB	7.15	3.19	
IL-15+TGFB	10.95	0.42	
**CD45RO+ , CCR7+**			
Ctrl	15.16	6.72	
TGFB	70.33	11.06	
IL-15+TGFB	70.58	0.50	
**CD45RO+ , CCR7-**			
Ctrl	70.30	11.64	
TGFB	20.40	9.94	
IL-15+TGFB	15.83	0.78	
**CD45RO- , CCR7-**			
Ctrl	10.17	5.15	
TGFB	2.16	1.58	
IL-15+TGFB	2.63	0.09	

OpTmizer	Mean	SD	Figure 3 Stats
**CD57-, KLRG1-**			
Ctrl	56.19	2.46	
TGFB	78.38	1.60	
IL-15+TGFB	78.85	4.51	
**CD57-, KLRG1+**			
Ctrl	27.34	14.43	
TGFB	14.90	1.19	
IL-15+TGFB	14.85	3.98	
**CD57+,KLRG1-**			
Ctrl	14.01	13.79	
TGFB	4.65	0.32	
IL-15+TGFB	4.39	0.09	
**CD57+, KLRG1+**			
Ctrl	2.47	0.68	
TGFB	2.11	0.30	
IL-15+TGFB	1.90	0.61	

Figure 3 Stats<br>Tukey's multiple comparisons test	RPMI + 10% FBS<br>Predicted (LS) mean diff.	95.00% CI of diff	Below threshold?	Summary	Adjusted P Value
**CD56-, CD16+**					
Ctrl vs. TGFB	18.15	11.45 to 24.84	Yes	****	<0.0001
Ctrl vs. IL15+TGFB	17.95	11.25 to 24.65	Yes	****	<0.0001
TGFB vs. IL15+TGFB	-0.1956	-6.893 to 6.502	No	ns	0.9973
**CD56+, CD16+**					
Ctrl vs. TGFB	21.3	14.60 to 27.99	Yes	****	<0.0001
Ctrl vs. IL15+TGFB	21.62	14.92 to 28.31	Yes	****	<0.0001
TGFB vs. IL15+TGFB	0.3189	-6.379 to 7.016	No	ns	0.9929
**CD56+, CD16-**					
Ctrl vs. TGFB	5.592	-1.105 to 12.29	No	ns	0.1207
Ctrl vs. IL15+TGFB	4.278	-2.420 to 10.98	No	ns	0.2859
TGFB vs. IL15+TGFB	-1.314	-8.012 to 5.383	No	ns	0.8868
**CD56-, CD16-**					
Ctrl vs. TGFB	-45.06	-51.75 to -38.36	Yes	****	<0.0001
Ctrl vs. IL15+TGFB	-43.87	-50.56 to -37.17	Yes	****	<0.0001
TGFB vs. IL15+TGFB	1.189	-5.509 to 7.886	No	ns	0.9064
**CD45RO- , CCR7+**					
Ctrl vs. TGFB	-8.473	-16.15 to -0.7993	Yes	*	0.0268
Ctrl vs. IL15+TGFB	-16.84	-24.52 to -9.168	Yes	****	<0.0001
TGFB vs. IL15+TGFB	-8.369	-16.04 to -0.6949	Yes	*	0.0291
**CD45RO+ , CCR7+**					
Ctrl vs. TGFB	-48.42	-56.10 to -40.75	Yes	****	<0.0001
Ctrl vs. IL15+TGFB	-45.95	-53.62 to -38.27	Yes	****	<0.0001
TGFB vs. IL15+TGFB	2.478	-5.196 to 10.15	No	ns	0.7231
**CD45RO+ , CCR7-**					
Ctrl vs. TGFB	54.56	46.88 to 62.23	Yes	****	<0.0001
Ctrl vs. IL15+TGFB	60.81	53.14 to 68.49	Yes	****	<0.0001
TGFB vs. IL15+TGFB	6.259	-1.415 to 13.93	No	ns	0.1327
**CD45RO- , CCR7-**					
Ctrl vs. TGFB	2.332	-5.342 to 10.01	No	ns	0.7502
Ctrl vs. IL15+TGFB	1.972	-5.702 to 9.646	No	ns	0.814
TGFB vs. IL15+TGFB	-0.36	-8.034 to 7.314	No	ns	0.9931

Figure 3 Stats<br>Tukey's multiple compariso ns test	RPMI + 10% FBS<br>Predicted (LS) mean diff.	95.00% CI of diff	Below threshold?	Summary	Adjusted P Value
**CD57-, KLRG1-**					
Ctrl vs. TGFB	-21.98	-31.53 to -12.42	Yes	****	<0.0001
Ctrl vs. IL15+TGFB	-24.04	-33.60 to -14.49	Yes	****	<0.0001
TGFB vs. IL15+TGFB	-2.067	-11.62 to 7.487	No	ns	0.8642
**CD57-, KLRG1+**					
Ctrl vs. TGFB	25.35	15.79 to 34.90	Yes	****	<0.0001
Ctrl vs. IL15+TGFB	26.84	17.29 to 36.40	Yes	****	<0.0001
TGFB vs. IL15+TGFB	1.497	-8.057 to 11.05	No	ns	0.9263
**CD57+,KLRG1-**					
Ctrl vs. TGFB	-5.4	-14.95 to 4.153	No	ns	0.3737
Ctrl vs. IL15+TGFB	-5.121	-14.67 to 4.432	No	ns	0.4121
TGFB vs. IL15+TGFB	0.2789	-9.274 to 9.832	No	ns	0.9973
**CD57+, KLRG1+**					
Ctrl vs. TGFB	2.773	-6.780 to 12.33	No	ns	0.7693
Ctrl vs. IL15+TGFB	3.09	-6.463 to 12.64	No	ns	0.7223
TGFB vs. IL15+TGFB	0.3167	-9.237 to 9.870	No	ns	0.9966

RPMI + 10% FBS	Mean	SD	Figure 3 Stats
**CD56-, CD16+**			
Ctrl	20.85	9.77	
TGFB	2.70	1.98	
IL-15+TGFB	2.90	1.95	
**CD56+, CD16+**			
Ctrl	23.22	7.39	
TGFB	1.93	2.24	
IL-15+TGFB	1.61	1.67	
**CD56+, CD16-**			
Ctrl	15.62	6.47	
TGFB	10.03	4.41	
IL-15+TGFB	11.34	1.98	
**CD56-, CD16-**			
Ctrl	40.29	9.96	
TGFB	85.34	8.31	
IL-15+TGFB	84.16	5.30	
**CD45RO- , CCR7+**			
Ctrl	1.71	0.91	
TGFB	10.19	7.17	
IL-15+TGFB	18.56	12.41	
**CD45RO+ , CCR7+**			
Ctrl	18.76	9.94	
TGFB	67.19	6.25	
IL-15+TGFB	64.71	8.51	
**CD45RO+ , CCR7-**			
Ctrl	75.86	6.87	
TGFB	21.30	8.16	
IL-15+TGFB	15.04	4.95	
**CD45RO- , CCR7-**			
Ctrl	3.65	2.37	
TGFB	1.32	0.69	
IL-15+TGFB	1.68	0.61	

RPMI + 10% FBS	Mean	SD	Figure 3 Stats
**CD57-, KLRG1-**			
Ctrl	60.06	16.43	
TGFB	82.03	4.66	
IL-15+TGFB	84.10	6.75	
**CD57-, KLRG1+**			
Ctrl	29.88	20.90	
TGFB	4.53	2.73	
IL-15+TGFB	3.04	1.91	
**CD57+,KLRG1-**			
Ctrl	6.88	6.25	
TGFB	12.28	3.40	
IL-15+TGFB	12.00	5.72	
**CD57+, KLRG1+**			
Ctrl	4.12	0.80	
TGFB	1.35	0.52	
IL-15+TGFB	1.03	0.60	

Figure 3 Stats<br>Tukey's multiple comparisons test	TheraPEAK Predicted (LS) mean diff.	95.00% CI of diff	Below threshold?	Summary	Adjusted P Value
**CD56-, CD16+**					
Ctrl vs. TGFB	14.11	3.528 to 24.69	Yes	**	0.0058
Ctrl vs. IL15+TGFB	14.51	3.933 to 25.09	Yes	**	0.0044
TGFB vs. IL15+TGFB	0.4044	-9.059 to 9.868	No	ns	0.9943
**CD56+, CD16+**					
Ctrl vs. TGFB	27.01	16.43 to 37.60	Yes	****	<0.0001
Ctrl vs. IL15+TGFB	27.37	16.79 to 37.95	Yes	****	<0.0001
TGFB vs. IL15+TGFB	0.3598	-9.104 to 9.823	No	ns	0.9955
**CD56+, CD16-**					
Ctrl vs. TGFB	7.878	-2.703 to 18.46	No	ns	0.1837
Ctrl vs. IL15+TGFB	2.39	-8.190 to 12.97	No	ns	0.8524
TGFB vs. IL15+TGFB	-5.488	-14.95 to 3.976	No	ns	0.354
**CD56-, CD16-**					
Ctrl vs. TGFB	-49	-59.58 to -38.42	Yes	****	<0.0001
Ctrl vs. IL15+TGFB	-44.29	-54.87 to -33.71	Yes	****	<0.0001
TGFB vs. IL15+TGFB	4.711	-4.752 to 14.17	No	ns	0.4637
**CD45RO- , CCR7+**					
Ctrl vs. TGFB	-7.25	-18.36 to 3.858	No	ns	0.2699
Ctrl vs. IL15+TGFB	-9.902	-21.01 to 1.206	No	ns	0.0905
TGFB vs. IL15+TGFB	-2.652	-12.59 to 7.283	No	ns	0.8002
**CD45RO+ , CCR7+**					
Ctrl vs. TGFB	-50.67	-61.78 to -39.57	Yes	****	<0.0001
Ctrl vs. IL15+TGFB	-58.4	-69.50 to -47.29	Yes	****	<0.0001
TGFB vs. IL15+TGFB	-7.722	-17.66 to 2.213	No	ns	0.1585
**CD45RO+ , CCR7-**					
Ctrl vs. TGFB	54.81	43.70 to 65.91	Yes	****	<0.0001
Ctrl vs. IL15+TGFB	65.65	54.54 to 76.75	Yes	****	<0.0001
TGFB vs. IL15+TGFB	10.84	0.9055 to 20.78	Yes	*	0.0291
**CD45RO- , CCR7-**					
Ctrl vs. TGFB	3.124	-7.984 to 14.23	No	ns	0.7809
Ctrl vs. IL15+TGFB	2.665	-8.443 to 13.77	No	ns	0.8352
TGFB vs. IL15+TGFB	-0.4589	-10.39 to 9.477	No	ns	0.9933

Figure 3 Stats<br>Tukey's multiple comparisons test	TheraPEAK<br>Predicted (LS) mean diff.	95.00% CI of diff	Below threshold?	Summary	Adjusted P Value
**CD57-, KLRG1-**					
Ctrl vs. TGFB	-36.23	-54.80 to -17.66	Yes	****	<0.0001
Ctrl vs. IL15+TGFB	-39.27	-57.84 to -20.70	Yes	****	<0.0001
TGFB vs. IL15+TGFB	-3.033	-19.64 to 13.58	No	ns	0.9008
**CD57-, KLRG1+**					
Ctrl vs. TGFB	31.05	12.48 to 49.62	Yes	***	0.0004
Ctrl vs. IL15+TGFB	34.29	15.71 to 52.86	Yes	****	<0.0001
TGFB vs. IL15+TGFB	3.237	-13.37 to 19.85	No	ns	0.8878
**CD57+,KLRG1-**					
Ctrl vs. TGFB	0.9478	-17.62 to 19.52	No	ns	0.9919
Ctrl vs. IL15+TGFB	0.5144	-18.06 to 19.09	No	ns	0.9976
TGFB vs. IL15+TGFB	-0.4333	-17.04 to 16.18	No	ns	0.9979
**CD57+, KLRG1+**					
Ctrl vs. TGFB	4.236	-14.33 to 22.81	No	ns	0.8497
Ctrl vs. IL15+TGFB	4.465	-14.11 to 23.04	No	ns	0.8345
TGFB vs. IL15+TGFB	0.2293	-16.38 to 16.84	No	ns	0.9994

TheraPEAK	Mean	SD	Figure 3 Stats
**CD56-, CD16+**			
Ctrl	16.00	5.45	
TGFB	1.89	1.65	
IL-15+TGFB	1.49	1.02	
**CD56+, CD16+**			
Ctrl	29.05	3.59	
TGFB	2.04	2.33	
IL-15+TGFB	1.68	1.75	
**CD56+, CD16-**			
Ctrl	22.78	3.91	
TGFB	14.91	10.63	
IL-15+TGFB	20.39	13.45	
**CD56-, CD16-**			
Ctrl	32.17	2.02	
TGFB	81.17	13.49	
IL-15+TGFB	76.46	14.71	
**CD45RO- , CCR7+**			
Ctrl	1.46	0.19	
TGFB	8.71	7.87	
IL-15+TGFB	11.36	11.51	
**CD45RO+ , CCR7+**			
Ctrl	9.63	6.79	
TGFB	60.30	8.27	
IL-15+TGFB	68.02	11.30	
**CD45RO+ , CCR7-**			
Ctrl	84.17	3.58	
TGFB	29.36	14.54	
IL-15+TGFB	18.52	13.07	
**CD45RO- , CCR7-**			
Ctrl	4.75	3.43	
TGFB	1.63	0.68	
IL-15+TGFB	2.09	1.39	

TheraPEAK	Mean	SD	Figure 3 Stats
**CD57-, KLRG1-**			
Ctrl	45.63	34.72	
TGFB	81.87	13.30	
IL-15+TGFB	84.90	9.23	
**CD57-, KLRG1+**			
Ctrl	41.00	38.51	
TGFB	9.95	12.94	
IL-15+TGFB	6.71	8.13	
**CD57+,KLRG1-**			
Ctrl	8.08	8.13	
TGFB	7.14	5.56	
IL-15+TGFB	7.57	5.31	
**CD57+, KLRG1+**			
Ctrl	5.28	4.34	
TGFB	1.04	1.12	
IL-15+TGFB	0.81	0.74	

Figure 3 Stats<br>Tukey's multiple comparisons test	TexMACS<br>Predicted (LS) mean diff.	95.00% CI of diff	Below threshold?	Summary	Adjusted P Value
**CD56-, CD16+**					
Ctrl vs. TGFB	9.929	2.150 to 17.71	Yes	**	0.0085
Ctrl vs. IL15+TGFB	9.866	2.086 to 17.64	Yes	**	0.009
TGFB vs. IL15+TGFB	-0.06333	-7.843 to 7.716	No	ns	0.9998
**CD56+, CD16+**					
Ctrl vs. TGFB	16.44	8.665 to 24.22	Yes	****	<0.0001
Ctrl vs. IL15+TGFB	16.49	8.711 to 24.27	Yes	****	<0.0001
TGFB vs. IL15+TGFB	0.04556	-7.734 to 7.825	No	ns	0.9999
**CD56+, CD16-**					
Ctrl vs. TGFB	7.562	-0.2171 to 15.34	No	ns	0.0586
Ctrl vs. IL15+TGFB	8.259	0.4796 to 16.04	Yes	*	0.0347
TGFB vs. IL15+TGFB	0.6967	-7.083 to 8.476	No	ns	0.9753
**CD56-, CD16-**					
Ctrl vs. TGFB	-33.96	-41.73 to -26.18	Yes	****	<0.0001
Ctrl vs. IL15+TGFB	-34.64	-42.42 to -26.87	Yes	****	<0.0001
TGFB vs. IL15+TGFB	-0.6889	-8.468 to 7.090	No	ns	0.9758
**CD45RO- , CCR7+**					
Ctrl vs. TGFB	-8.337	-23.94 to 7.268	No	ns	0.4133
Ctrl vs. IL15+TGFB	-12.12	-27.72 to 3.489	No	ns	0.1591
TGFB vs. IL15+TGFB	-3.779	-17.74 to 10.18	No	ns	0.7951
**CD45RO+ , CCR7+**					
Ctrl vs. TGFB	-40.81	-56.41 to -25.20	Yes	****	<0.0001
Ctrl vs. IL15+TGFB	-42.18	-57.78 to -26.57	Yes	****	<0.0001
TGFB vs. IL15+TGFB	-1.367	-15.32 to 12.59	No	ns	0.9704
**CD45RO+ , CCR7-**					
Ctrl vs. TGFB	44.65	29.05 to 60.26	Yes	****	<0.0001
Ctrl vs. IL15+TGFB	50	34.39 to 65.60	Yes	****	<0.0001
TGFB vs. IL15+TGFB	5.346	-8.612 to 19.30	No	ns	0.6331
**CD45RO- , CCR7-**					
Ctrl vs. TGFB	4.533	-11.07 to 20.14	No	ns	0.7681
Ctrl vs. IL15+TGFB	4.318	-11.29 to 19.92	No	ns	0.7871
TGFB vs. IL15+TGFB	-0.2156	-14.17 to 13.74	No	ns	0.9993

Figure 3 Stats<br>Tukey's multiple comparisons test	TexMACS<br>Predicted (LS) mean diff.	95.00% CI of diff	Below threshold?	Summary	Adjusted P Value
**CD57-, KLRG1-**					
Ctrl vs. TGFB	-29.18	-43.73 to -14.63	Yes	****	<0.0001
Ctrl vs. IL15+TGFB	-32.48	-47.03 to -17.93	Yes	****	<0.0001
TGFB vs. IL15+TGFB	-3.3	-16.32 to 9.715	No	ns	0.8178
**CD57-, KLRG1+**					
Ctrl vs. TGFB	22.48	7.932 to 37.03	Yes	**	0.0012
Ctrl vs. IL15+TGFB	24.93	10.38 to 39.49	Yes	***	0.0003
TGFB vs. IL15+TGFB	2.451	-10.56 to 15.47	No	ns	0.8948
**CD57+,KLRG1-**					
Ctrl vs. TGFB	1.646	-12.91 to 16.20	No	ns	0.9607
Ctrl vs. IL15+TGFB	2.204	-12.35 to 16.76	No	ns	0.9306
TGFB vs. IL15+TGFB	0.5589	-12.46 to 13.57	No	ns	0.9942
**CD57+, KLRG1+**					
Ctrl vs. TGFB	5.056	-9.496 to 19.61	No	ns	0.6862
Ctrl vs. IL15+TGFB	5.371	-9.180 to 19.92	No	ns	0.6539
TGFB vs. IL15+TGFB	0.3156	-12.70 to 13.33	No	ns	0.9982

TexMACS	Mean	SD	Figure 3 Stats
**CD56-, CD16+**			
Ctrl	11.78	6.98	
TGFB	1.85	0.65	
IL-15+TGFB	1.91	0.98	
**CD56+, CD16+**			
Ctrl	17.88	3.59	
TGFB	1.43	1.35	
IL-15+TGFB	1.39	1.22	
**CD56+, CD16-**			
Ctrl	17.73	5.09	
TGFB	10.17	9.18	
IL-15+TGFB	9.47	7.14	
**CD56-, CD16-**			
Ctrl	52.60	11.96	
TGFB	86.56	11.12	
IL-15+TGFB	87.24	9.08	
**CD45RO- , CCR7+**			
Ctrl	2.95	0.74	
TGFB	11.46	2.68	
IL-15+TGFB	15.60	3.37	
**CD45RO+ , CCR7+**			
Ctrl	12.63	12.35	
TGFB	58.19	21.02	
IL-15+TGFB	59.21	17.21	
**CD45RO+ , CCR7-**			
Ctrl	76.80	9.55	
TGFB	28.33	21.07	
IL-15+TGFB	22.77	16.02	
**CD45RO- , CCR7-**			
Ctrl	7.65	3.52	
TGFB	2.01	1.48	
IL-15+TGFB	2.41	1.29	

TexMACS	Mean	SD	Figure 3 Stats
**CD57-, KLRG1-**			
Ctrl	53.78	27.77	
TGFB	82.97	8.48	
IL-15+TGFB	86.27	5.63	
**CD57-, KLRG1+**			
Ctrl	31.30	30.24	
TGFB	8.81	10.08	
IL-15+TGFB	6.36	7.34	
**CD57+,KLRG1-**			
Ctrl	8.97	7.70	
TGFB	7.33	4.82	
IL-15+TGFB	6.77	3.92	
**CD57+, KLRG1+**			
Ctrl	5.96	5.23	
TGFB	0.91	0.72	
IL-15+TGFB	0.59	0.43	

Figure 4 **Blood 144h**

Tukey's multiple comparisons test	Mean Diff.	95.00% CI of diff.	Below threshold?	Summary	Adjusted P Value
Control vs. TGF-B	-11.87	-17.58 to -6.161	Yes	***	0.0002
Control vs. TGF-B+IL-15	-11.27	-17.28 to -5.248	Yes	***	0.0005
Control vs. Frozen TGF-B	-9.09	-15.11 to -3.073	Yes	**	0.0033
TGF-B vs. TGF-B+IL-15	0.605	-5.104 to 6.314	No	ns	0.9891
TGF-B vs. Frozen TGF-B	2.78	-2.929 to 8.489	No	ns	0.5043
TGF-B+IL-15 vs. Frozen TGF-B	2.175	-3.842 to 8.192	No	ns	0.7181

Test details	Mean 1	Mean 2	Mean Diff.
Control vs. TGF-B	5.31	17.18	-11.87
Control vs. TGF-B+IL-15	5.31	16.58	-11.27
Control vs. Frozen TGF-B	5.31	14.4	-9.09
TGF-B vs. TGF-B+IL-15	17.18	16.58	0.605
TGF-B vs. Frozen TGF-B	17.18	14.4	2.78
TGF-B+IL-15 vs. Frozen TGF-B	16.58	14.4	2.175

	SE of diff.	n1	n2	q	DF
Control vs. TGF-B	1.945	4	5	8.631	13
Control vs. TGF-B+IL-15	2.05	4	4	7.771	13
Control vs. Frozen TGF-B	2.05	4	4	6.27	13
TGF-B vs. TGF-B+IL-15	1.945	5	4	0.4399	13
TGF-B vs. Frozen TGF-B	1.945	5	4	2.021	13
TGF-B+IL-15 vs. Frozen TGF-B	2.05	4	4	1.5	13

Figure 4 **Bone Marrow 144h**

Tukey's multiple comparisons test	Mean Diff.	95.00% CI of diff.	Below threshold?	Summary	Adjusted P Value
Control vs. TGF-B	-0.5045	-0.6463 to -0.3627	Yes	****	<0.0001
Control vs. TGF-B+IL-15	-0.4775	-0.6270 to -0.3280	Yes	****	<0.0001
Control vs. Frozen TGF-B	-0.25	-0.3995 to -0.1005	Yes	***	0.0009
TGF-B vs. TGF-B+IL-15	0.027	-0.1148 to 0.1688	No	ns	0.9749
TGF-B vs. Frozen TGF-B	0.2545	0.1127 to 0.3963	Yes	***	0.0005
TGF-B+IL-15 vs. Frozen TGF-B	0.2275	0.07799 to 0.3770	Yes	**	0.0023

Test details	Mean 1	Mean 2	Mean Diff.
Control vs. TGF-B	0.0775	0.582	-0.5045
Control vs. TGF-B+IL-15	0.0775	0.555	-0.4775
Control vs. Frozen TGF-B	0.0775	0.3275	-0.25
TGF-B vs. TGF-B+IL-15	0.582	0.555	0.027
TGF-B vs. Frozen TGF-B	0.582	0.3275	0.2545
TGF-B+IL-15 vs. Frozen TGF-B	0.555	0.3275	0.2275

	SE of diff.	n1	n2	q	DF
Control vs. TGF-B	0.04593	4	5	15.53	15
Control vs. TGF-B+IL-15	0.04842	4	4	13.95	15
Control vs. Frozen TGF-B	0.04842	4	4	7.302	15
TGF-B vs. TGF-B+IL-15	0.04593	5	4	0.8313	15
TGF-B vs. Frozen TGF-B	0.04593	5	4	7.836	15
TGF-B+IL-15 vs. Frozen TGF-B	0.04842	4	4	6.645	15

Figure 5 **Comparison of Survival Curves**

**Log-rank (Mantel-Cox) test**		
Chi square	16.24	
df	1	
P value	<0.0001	
P value summary	****	
Are the survival curves sig different?	Yes	
**Median survival**		
**Untreated Mice**	21	
**TGFB + Tx**	47	
Ratio (and its reciprocal)	0.4468	2.238
95% CI of ratio	0.1717 to 1.163	0.8601 to 5.824

**Log-rank (Mantel-Cox) test**		
Chi square	11.14	
df	1	
P value	0.0008	
P value summary	***	
Are the survival curves sig different?	Yes	
**Median survival**		
**200mg/kg IFO + 3mg Zol**	32	
**TGFB + Tx**	47	
Ratio (and its reciprocal)	0.6809	1.469
95% CI of ratio	0.2078 to 2.231	0.4482 to 4.813

**Figure 5**	**Comparison of Survival Curves**	
**Log-rank (Mantel-Cox) test**		
Chi square	11.46	
df	1	
P value	0.0007	
P value summary	***	
Are the survival curves sig different?	Yes	
**Median survival**		
**Gamma Delta Alone**	29	
**TGFB + Tx**	47	
Ratio (and its reciprocal)	0.617	1.621
95% CI of ratio	0.1883 to 2.022	0.4946 to 5.311

**Log-rank (Mantel-Cox) test**		
Chi square	10.57	
df	1	
P value	0.0011	
P value summary	**	
Are the survival curves sig different?	Yes	
**Median survival**		
**Control + Tx**	33	
**TGFB + Tx**	47	
Ratio (and its reciprocal)	0.7021	1.424
95% CI of ratio	0.2143 to 2.301	0.4347 to 4.667

Supplemental Figure 2 **Comparison of Survival Curves**

**Log-rank (Mantel-Cox) test**		
Chi square	5.582	
df	1	
P value	0.0181	
P value summary	*	
Are the survival curves sig different?	Yes	
**Median survival**		
**Control Mice**	21	
**Non-Mod gd**	27	
Ratio (and its reciprocal)	0.7778	1.286
95% CI of ratio	0.2106 to 2.873	0.3481 to 4.749

**Log-rank (Mantel-Cox) test**		
Chi square	5.049	
df	1	
P value	0.0246	
P value summary	*	
Are the survival curves sig different?	Yes	
**Median survival**		
**Control Mice**	21	
**Non-Mod + Zol**	28	
Ratio (and its reciprocal)	0.75	1.333
95% CI of ratio	0.2030 to 2.770	0.3610 to 4.925

Supplemental Figure 4

Tukey's multiple comparisons test	OpTmizer Mean Diff.	95.00% CI of diff.	Below threshold?	Summary	Adjusted P Value
**CD27+,CD62L+**					
Ctrl vs. TGFB	-7.567	-16.95 to 1.819	No	ns	0.1388
Ctrl vs. IL15+TGFB	-4.456	-13.84 to 4.930	No	ns	0.498
TGFB vs. IL15+TGFB	3.111	-6.275 to 12.50	No	ns	0.7106
**CD27-,CD62L+**					
Ctrl vs. TGFB	-4.806	-14.19 to 4.580	No	ns	0.445
Ctrl vs. IL15+TGFB	-3.693	-13.08 to 5.692	No	ns	0.6185
TGFB vs. IL15+TGFB	1.112	-8.273 to 10.50	No	ns	0.9571
**CD27+,CD62L-**					
Ctrl vs. TGFB	0.5	-8.886 to 9.886	No	ns	0.9912
Ctrl vs. IL15+TGFB	-0.08889	-9.475 to 9.297	No	ns	0.9997
TGFB vs. IL15+TGFB	-0.5889	-9.975 to 8.797	No	ns	0.9878
**CD27-,CD62L-**					
Ctrl vs. TGFB	11.12	1.737 to 20.51	Yes	*	0.0159
Ctrl vs. IL15+TGFB	8.956	-0.4302 to 18.34	No	ns	0.0648
TGFB vs. IL15+TGFB	-2.167	-11.55 to 7.219	No	ns	0.847

OpTmizer	Mean	SD
**CD27+,CD62L+**		
Ctrl	19.97	2.23
TGFB	27.53	12.14
IL-15+TGFB	24.42	2.80
**CD27-,CD62L+**		
Ctrl	10.26	8.59
TGFB	15.07	3.70
IL-15+TGFB	13.95	7.24
**CD27+,CD62L-**		
Ctrl	16.87	5.04
TGFB	16.37	5.71
IL-15+TGFB	16.96	3.18
**CD27-,CD62L-**		
Ctrl	52.91	10.52
TGFB	41.79	16.62
IL-15+TGFB	43.96	9.17

Supplemental Figure 4

Tukey's multiple comparisons test	RPMI + 10% FBS Mean Diff.	95.00% CI of diff.	Below threshold?	Summary	Adjusted P Value
**CD27+,CD62L+**					
Ctrl vs. TGFB	-3.17	-8.358 to 2.018	No	ns	0.3174
Ctrl vs. IL15+TGFB	-4.59	-9.778 to 0.5984	No	ns	0.0939
TGFB vs. IL15+TGFB	-1.42	-6.608 to 3.768	No	ns	0.7919
**CD27-,CD62L+**					
Ctrl vs. TGFB	-0.2378	-5.426 to 4.951	No	ns	0.9935
Ctrl vs. IL15+TGFB	5.976	0.7872 to 11.16	Yes	*	0.0198
TGFB vs. IL15+TGFB	6.213	1.025 to 11.40	Yes	*	0.0146
**CD27+,CD62L-**					
Ctrl vs. TGFB	5.746	0.5572 to 10.93	Yes	*	0.0262
Ctrl vs. IL15+TGFB	4.219	-0.9695 to 9.407	No	ns	0.1342
TGFB vs. IL15+TGFB	-1.527	-6.715 to 3.662	No	ns	0.7638
**CD27-,CD62L-**					
Ctrl vs. TGFB	-2.344	-7.533 to 2.844	No	ns	0.5314
Ctrl vs. IL15+TGFB	-5.6	-10.79 to -0.4116	Yes	*	0.0312
TGFB vs. IL15+TGFB	-3.256	-8.444 to 1.933	No	ns	0.2984

RPMI + 10% FBS

	Mean	SD
**CD27+,CD62L+**		
Ctrl	6.22	0.76
TGFB	9.39	2.57
IL-15+TGFB	10.81	5.76
**CD27-,CD62L+**		
Ctrl	12.81	6.06
TGFB	13.05	4.30
IL-15+TGFB	6.83	1.84
**CD27+,CD62L-**		
Ctrl	17.36	1.37
TGFB	11.61	3.92
IL-15+TGFB	13.14	2.93
**CD27-,CD62L-**		
Ctrl	63.61	6.37
TGFB	65.96	3.30
IL-15+TGFB	69.21	8.96

Supplemental Figure 4

Tukey's multiple comparisons test	Mean Diff.	95.00% CI of diff.	Below threshold?	Summary	Adjusted P Value
**CD27+,CD62L+**					
Ctrl vs. TGFB	-9.568	-24.10 to 4.964	No	ns	0.2647
Ctrl vs. IL15+TGFB	-5.361	-19.89 to 9.170	No	ns	0.6553
TGFB vs. IL15+TGFB	4.207	-10.32 to 18.74	No	ns	0.7704
**CD27-,CD62L+**					
Ctrl vs. TGFB	-5.363	-19.89 to 9.168	No	ns	0.655
Ctrl vs. IL15+TGFB	-10.22	-24.75 to 4.308	No	ns	0.2201
TGFB vs. IL15+TGFB	-4.86	-19.39 to 9.672	No	ns	0.7063
**CD27+,CD62L-**					
Ctrl vs. TGFB	-5.818	-20.35 to 8.714	No	ns	0.6082
Ctrl vs. IL15+TGFB	-4.364	-18.90 to 10.17	No	ns	0.7552
TGFB vs. IL15+TGFB	1.453	-13.08 to 15.98	No	ns	0.9692
**CD27-,CD62L-**					
Ctrl vs. TGFB	22.17	7.635 to 36.70	Yes	**	0.0013
Ctrl vs. IL15+TGFB	20.21	5.680 to 34.74	Yes	**	0.0037
TGFB vs. IL15+TGFB	-1.956	-16.49 to 12.58	No	ns	0.945

Table heading: **TheraPEAK**

TheraPEAK

	Mean	SD
**CD27+,CD62L+**		
Ctrl	6.78	3.25
TGFB	16.35	7.38
IL-15+TGFB	12.14	1.38
**CD27-,CD62L+**		
Ctrl	14.68	9.88
TGFB	20.05	15.75
IL-15+TGFB	24.91	26.27
**CD27+,CD62L-**		
Ctrl	11.80	4.04
TGFB	17.61	7.29
IL-15+TGFB	16.16	9.87
**CD27-,CD62L-**		
Ctrl	68.58	10.47
TGFB	46.41	14.26
IL-15+TGFB	48.37	20.73

Supplemental Figure 4 **TexMACS**

Tukey's multiple comparisons test	Mean Diff.	95.00% CI of diff.	Below threshold?	Summary	Adjusted P Value
**CD27+,CD62L+**					
Ctrl vs. TGFB	-6.722	-16.03 to 2.588	No	ns	0.2034
Ctrl vs. IL15+TGFB	-3.4	-12.71 to 5.910	No	ns	0.6608
TGFB vs. IL15+TGFB	3.322	-5.988 to 12.63	No	ns	0.6732
**CD27-,CD62L+**					
Ctrl vs. TGFB	12.47	3.161 to 21.78	Yes	**	0.0054
Ctrl vs. IL15+TGFB	15.34	6.032 to 24.65	Yes	***	0.0005
TGFB vs. IL15+TGFB	2.871	-6.439 to 12.18	No	ns	0.7438
**CD27+,CD62L-**					
Ctrl vs. TGFB	-0.5333	-9.843 to 8.776	No	ns	0.9898
Ctrl vs. IL15+TGFB	-1.756	-11.07 to 7.554	No	ns	0.895
TGFB vs. IL15+TGFB	-1.222	-10.53 to 8.088	No	ns	0.9476
**CD27-,CD62L-**					
Ctrl vs. TGFB	-5.3	-14.61 to 4.010	No	ns	0.3685
Ctrl vs. IL15+TGFB	-10.21	-19.52 to -0.9014	Yes	*	0.028
TGFB vs. IL15+TGFB	-4.911	-14.22 to 4.399	No	ns	0.4236

TexMACS

	Mean	SD
**CD27+,CD62L+**		
Ctrl	12.47	8.30
TGFB	19.19	11.63
IL-15+TGFB	15.87	4.35
**CD27-,CD62L+**	22.88	8.22
Ctrl	10.41	2.96
TGFB	7.54	2.62
IL-15+TGFB		
**CD27+,CD62L-**	13.16	3.49
Ctrl	13.69	1.23
TGFB	14.91	1.65
IL-15+TGFB		
**CD27-,CD62L-**		
Ctrl	51.46	16.26
TGFB	56.76	14.07
IL-15+TGFB	61.67	6.39

Supplemental Figure 6

Tukey's multiple comparisons test	Mean Diff.	95.00% CI of diff.	Below threshold?	Summary	Adjusted P Value
**CD45RO- , CCR7+**					
**Ctrl** vs. Ctrl 1:1	-3.307	-9.226 to 2.613	No	ns	0.5654
**Ctrl** vs. Ctrl 5:1	-1.14	-7.059 to 4.779	No	ns	0.9924
**Ctrl** vs. **TGFB**	-11.61	-17.53 to -5.691	Yes	****	<0.0001
**Ctrl** vs. TGFB 1:1	-21.48	-27.40 to -15.56	Yes	****	<0.0001
**Ctrl** vs. TGFB 5:1	-21.74	-27.66 to -15.82	Yes	****	<0.0001
Ctrl 1:1 vs. Ctrl 5:1	2.167	-3.753 to 8.086	No	ns	0.8844
Ctrl 1:1 vs. **TGFB**	-8.303	-14.22 to -2.384	Yes	**	0.0017
Ctrl 1:1 vs. TGFB 1:1	-18.17	-24.09 to -12.25	Yes	****	<0.0001
Ctrl 1:1 vs. TGFB 5:1	-18.44	-24.36 to -12.52	Yes	****	<0.0001
Ctrl 5:1 vs. **TGFB**	-10.47	-16.39 to -4.551	Yes	****	<0.0001
Ctrl 5:1 vs. TGFB 1:1	-20.34	-26.26 to -14.42	Yes	****	<0.0001
Ctrl 5:1 vs. TGFB 5:1	-20.6	-26.52 to -14.68	Yes	****	<0.0001
**TGFB** vs. TGFB 1:1	-9.867	-15.79 to -3.947	Yes	***	0.0001
**TGFB** vs. TGFB 5:1	-10.13	-16.05 to -4.214	Yes	****	<0.0001
TGFB 1:1 vs. TGFB 5:1	-0.2667	-6.186 to 5.653	No	ns	>0.9999
**CD45RO+ , CCR7+**					
**Ctrl** vs. Ctrl 1:1	3.713	-2.206 to 9.633	No	ns	0.4375
**Ctrl** vs. Ctrl 5:1	3.07	-2.849 to 8.989	No	ns	0.6412
**Ctrl** vs. **TGFB**	-16.19	-22.11 to -10.27	Yes	****	<0.0001
**Ctrl** vs. TGFB 1:1	-5.193	-11.11 to 0.7260	No	ns	0.116
**Ctrl** vs. TGFB 5:1	-4.727	-10.65 to 1.193	No	ns	0.1875
Ctrl 1:1 vs. Ctrl 5:1	-0.6433	-6.563 to 5.276	No	ns	0.9995
Ctrl 1:1 vs. **TGFB**	-19.91	-25.83 to -13.99	Yes	****	<0.0001
Ctrl 1:1 vs. TGFB 1:1	-8.907	-14.83 to -2.987	Yes	***	0.0007
Ctrl 1:1 vs. TGFB 5:1	-8.44	-14.36 to -2.521	Yes	**	0.0014
Ctrl 5:1 vs. **TGFB**	-19.26	-25.18 to -13.34	Yes	****	<0.0001
Ctrl 5:1 vs. TGFB 1:1	-8.263	-14.18 to -2.344	Yes	**	0.0018
Ctrl 5:1 vs. TGFB 5:1	-7.797	-13.72 to -1.877	Yes	**	0.0037
**TGFB** vs. TGFB 1:1	11	5.081 to 16.92	Yes	****	<0.0001
**TGFB** vs. TGFB 5:1	11.47	5.547 to 17.39	Yes	****	<0.0001
TGFB 1:1 vs. TGFB 5:1	0.4667	-5.453 to 6.386	No	ns	0.9999

Supplemental Figure 6

Tukey's multiple comparisons test	Mean Diff.	95.00% CI of diff.	Below threshold?	Summary	Adjusted P Value
**CD45RO+ , CCR7-**					
**Ctrl** vs. Ctrl 1:1	8.667	2.747 to 14.59	Yes	***	0.001
**Ctrl** vs. Ctrl 5:1	2.7	-3.219 to 8.619	No	ns	0.7537
**Ctrl** vs. **TGFB**	33.1	27.18 to 39.02	Yes	****	<0.0001
**Ctrl** vs. TGFB 1:1	40.63	34.71 to 46.55	Yes	****	<0.0001
**Ctrl** vs. TGFB 5:1	43.23	37.31 to 49.15	Yes	****	<0.0001
Ctrl 1:1 vs. Ctrl 5:1	-5.967	-11.89 to -0.04733	Yes	*	0.0472
Ctrl 1:1 vs. **TGFB**	24.43	18.51 to 30.35	Yes	****	<0.0001
Ctrl 1:1 vs. TGFB 1:1	31.97	26.05 to 37.89	Yes	****	<0.0001
Ctrl 1:1 vs. TGFB 5:1	34.57	28.65 to 40.49	Yes	****	<0.0001
Ctrl 5:1 vs. **TGFB**	30.4	24.48 to 36.32	Yes	****	<0.0001
Ctrl 5:1 vs. TGFB 1:1	37.93	32.01 to 43.85	Yes	****	<0.0001
Ctrl 5:1 vs. TGFB 5:1	40.53	34.61 to 46.45	Yes	****	<0.0001
**TGFB** vs. TGFB 1:1	7.533	1.614 to 13.45	Yes	**	0.0055
**TGFB** vs. TGFB 5:1	10.13	4.214 to 16.05	Yes	****	<0.0001
TGFB 1:1 vs. TGFB 5:1	2.6	-3.319 to 8.519	No	ns	0.7816
**CD45RO- , CCR7-**					
**Ctrl** vs. Ctrl 1:1	-9.07	-14.99 to -3.151	Yes	***	0.0005
**Ctrl** vs. Ctrl 5:1	-4.603	-10.52 to 1.316	No	ns	0.2109
**Ctrl** vs. **TGFB**	-5.303	-11.22 to 0.6160	No	ns	0.1029
**Ctrl** vs. TGFB 1:1	-14.04	-19.96 to -8.117	Yes	****	<0.0001
**Ctrl** vs. TGFB 5:1	-16.77	-22.69 to -10.85	Yes	****	<0.0001
Ctrl 1:1 vs. Ctrl 5:1	4.467	-1.453 to 10.39	No	ns	0.2393
Ctrl 1:1 vs. **TGFB**	3.767	-2.153 to 9.686	No	ns	0.4214
Ctrl 1:1 vs. TGFB 1:1	-4.967	-10.89 to 0.9527	No	ns	0.1474
Ctrl 1:1 vs. TGFB 5:1	-7.7	-13.62 to -1.781	Yes	**	0.0043
Ctrl 5:1 vs. **TGFB**	-0.7	-6.619 to 5.219	No	ns	0.9993
Ctrl 5:1 vs. TGFB 1:1	-9.433	-15.35 to -3.514	Yes	***	0.0003
Ctrl 5:1 vs. TGFB 5:1	-12.17	-18.09 to -6.247	Yes	****	<0.0001
**TGFB** vs. TGFB 1:1	-8.733	-14.65 to -2.814	Yes	***	0.0009
**TGFB** vs. TGFB 5:1	-11.47	-17.39 to -5.547	Yes	****	<0.0001
TGFB 1:1 vs. TGFB 5:1	-2.733	-8.653 to 3.186	No	ns	0.7441

Supplemental Figure 6

Tukey's multiple comparisons test	Mean Diff.	95.00% CI of diff.	Below threshold?	Summary	Adjusted P Value	
**CD56-, CD16+**						
**Ctrl** vs. Ctrl 1:1	-2.333	-5.579 to 0.9120	No	ns		0.2878
**Ctrl** vs. Ctrl 5:1	-2	-5.245 to 1.245	No	ns		0.4575
**Ctrl** vs. **TGFB**	19.58	16.33 to 22.83	Yes	****	<0.0001	
**Ctrl** vs. TGFB 1:1	15.64	12.40 to 18.89	Yes	****	<0.0001	
**Ctrl** vs. TGFB 5:1	19.49	16.24 to 22.73	Yes	****	<0.0001	
Ctrl 1:1 vs. Ctrl 5:1	0.3333	-2.912 to 3.579	No	ns		0.9996
Ctrl 1:1 vs. **TGFB**	21.91	18.67 to 25.16	Yes	****	<0.0001	
Ctrl 1:1 vs. TGFB 1:1	17.98	14.73 to 21.22	Yes	****	<0.0001	
Ctrl 1:1 vs. TGFB 5:1	21.82	18.57 to 25.07	Yes	****	<0.0001	
Ctrl 5:1 vs. **TGFB**	21.58	18.33 to 24.83	Yes	****	<0.0001	
Ctrl 5:1 vs. TGFB 1:1	17.64	14.40 to 20.89	Yes	****	<0.0001	
Ctrl 5:1 vs. TGFB 5:1	21.49	18.24 to 24.73	Yes	****	<0.0001	
**TGFB** vs. TGFB 1:1	-3.937	-7.182 to -0.6914	Yes	**		0.0092
**TGFB** vs. TGFB 5:1	-0.09333	-3.339 to 3.152	No	ns	>0.9999	
TGFB 1:1 vs. TGFB 5:1	3.843	0.5980 to 7.089	Yes	*		0.0118
**CD56+, CD16+**						
**Ctrl** vs. Ctrl 1:1	4.3	1.055 to 7.545	Yes	**		0.0035
**Ctrl** vs. Ctrl 5:1	2.267	-0.9786 to 5.512	No	ns		0.3185
**Ctrl** vs. **TGFB**	23.58	20.34 to 26.83	Yes	****	<0.0001	
**Ctrl** vs. TGFB 1:1	22.54	19.29 to 25.79	Yes	****	<0.0001	
**Ctrl** vs. TGFB 5:1	24.31	21.06 to 27.55	Yes	****	<0.0001	
Ctrl 1:1 vs. Ctrl 5:1	-2.033	-5.279 to 1.212	No	ns		0.4389
Ctrl 1:1 vs. **TGFB**	19.28	16.04 to 22.53	Yes	****	<0.0001	
Ctrl 1:1 vs. TGFB 1:1	18.24	14.99 to 21.49	Yes	****	<0.0001	
Ctrl 1:1 vs. TGFB 5:1	20.01	16.76 to 23.25	Yes	****	<0.0001	
Ctrl 5:1 vs. **TGFB**	21.32	18.07 to 24.56	Yes	****	<0.0001	
Ctrl 5:1 vs. TGFB 1:1	20.27	17.03 to 23.52	Yes	****	<0.0001	
Ctrl 5:1 vs. TGFB 5:1	22.04	18.79 to 25.29	Yes	****	<0.0001	
**TGFB** vs. TGFB 1:1	-1.043	-4.289 to 2.202	No	ns		0.9299
**TGFB** vs. TGFB 5:1	0.7233	-2.522 to 3.969	No	ns		0.9853
TGFB 1:1 vs. TGFB 5:1	1.767	-1.479 to 5.012	No	ns		0.5925

Supplemental Figure 6

Tukey's multiple comparisons test	Mean Diff.	95.00% CI of diff.	Below threshold?	Summary	Adjusted P Value
**CD56+, CD16-**					
**Ctrl** vs. Ctrl 1:1	2.467	-0.7786 to 5.712	No	ns	0.2323
**Ctrl** vs. Ctrl 5:1	2.133	-1.112 to 5.379	No	ns	0.385
**Ctrl** vs. **TGFB**	-16.9	-20.15 to -13.65	Yes	****	<0.0001
**Ctrl** vs. TGFB 1:1	-7.033	-10.28 to -3.788	Yes	****	<0.0001
**Ctrl** vs. TGFB 5:1	-11.6	-14.85 to -8.355	Yes	****	<0.0001
Ctrl 1:1 vs. Ctrl 5:1	-0.3333	-3.579 to 2.912	No	ns	0.9996
Ctrl 1:1 vs. **TGFB**	-19.37	-22.61 to -16.12	Yes	****	<0.0001
Ctrl 1:1 vs. TGFB 1:1	-9.5	-12.75 to -6.255	Yes	****	<0.0001
Ctrl 1:1 vs. TGFB 5:1	-14.07	-17.31 to -10.82	Yes	****	<0.0001
Ctrl 5:1 vs. **TGFB**	-19.03	-22.28 to -15.79	Yes	****	<0.0001
Ctrl 5:1 vs. TGFB 1:1	-9.167	-12.41 to -5.921	Yes	****	<0.0001
Ctrl 5:1 vs. TGFB 5:1	-13.73	-16.98 to -10.49	Yes	****	<0.0001
**TGFB** vs. TGFB 1:1	9.867	6.621 to 13.11	Yes	****	<0.0001
**TGFB** vs. TGFB 5:1	5.3	2.055 to 8.545	Yes	***	0.0002
TGFB 1:1 vs. TGFB 5:1	-4.567	-7.812 to -1.321	Yes	**	0.0016
**CD56-, CD16-**					
**Ctrl** vs. Ctrl 1:1	-4.5	-7.745 to -1.255	Yes	**	0.002
**Ctrl** vs. Ctrl 5:1	-2.4	-5.645 to 0.8453	No	ns	0.2591
**Ctrl** vs. **TGFB**	-26.27	-29.51 to -23.02	Yes	****	<0.0001
**Ctrl** vs. TGFB 1:1	-31.17	-34.41 to -27.92	Yes	****	<0.0001
**Ctrl** vs. TGFB 5:1	-32.2	-35.45 to -28.95	Yes	****	<0.0001
Ctrl 1:1 vs. Ctrl 5:1	2.1	-1.145 to 5.345	No	ns	0.4026
Ctrl 1:1 vs. **TGFB**	-21.77	-25.01 to -18.52	Yes	****	<0.0001
Ctrl 1:1 vs. TGFB 1:1	-26.67	-29.91 to -23.42	Yes	****	<0.0001
Ctrl 1:1 vs. TGFB 5:1	-27.7	-30.95 to -24.45	Yes	****	<0.0001
Ctrl 5:1 vs. **TGFB**	-23.87	-27.11 to -20.62	Yes	****	<0.0001
Ctrl 5:1 vs. TGFB 1:1	-28.77	-32.01 to -25.52	Yes	****	<0.0001
Ctrl 5:1 vs. TGFB 5:1	-29.8	-33.05 to -26.55	Yes	****	<0.0001
**TGFB** vs. TGFB 1:1	-4.9	-8.145 to -1.655	Yes	***	0.0006
**TGFB** vs. TGFB 5:1	-5.933	-9.179 to -2.688	Yes	****	<0.0001
TGFB 1:1 vs. TGFB 5:1	-1.033	-4.279 to 2.212	No	ns	0.9325

Supplemental Figure 6

Tukey's multiple comparisons test	Mean Diff.	95.00% CI of diff.	Below threshold?	Summary	Adjusted P Value
**CD27-, CD62L+**					
**Ctrl** vs. Ctrl 1:1	1.47	-2.751 to 5.691	No	ns	0.9042
**Ctrl** vs. Ctrl 5:1	2.53	-1.691 to 6.751	No	ns	0.4886
**Ctrl** vs. **TGFB**	-8.903	-13.12 to -4.682	Yes	****	<0.0001
**Ctrl** vs. TGFB 1:1	-9.003	-13.22 to -4.782	Yes	****	<0.0001
**Ctrl** vs. TGFB 5:1	-6.97	-11.19 to -2.749	Yes	***	0.0002
Ctrl 1:1 vs. Ctrl 5:1	1.06	-3.161 to 5.281	No	ns	0.975
Ctrl 1:1 vs. **TGFB**	-10.37	-14.59 to -6.152	Yes	****	<0.0001
Ctrl 1:1 vs. TGFB 1:1	-10.47	-14.69 to -6.252	Yes	****	<0.0001
Ctrl 1:1 vs. TGFB 5:1	-8.44	-12.66 to -4.219	Yes	****	<0.0001
Ctrl 5:1 vs. **TGFB**	-11.43	-15.65 to -7.212	Yes	****	<0.0001
Ctrl 5:1 vs. TGFB 1:1	-11.53	-15.75 to -7.312	Yes	****	<0.0001
Ctrl 5:1 vs. TGFB 5:1	-9.5	-13.72 to -5.279	Yes	****	<0.0001
**TGFB** vs. TGFB 1:1	-0.1	-4.321 to 4.121	No	ns	>0.9999
**TGFB** vs. TGFB 5:1	1.933	-2.288 to 6.154	No	ns	0.7504
TGFB 1:1 vs. TGFB 5:1	2.033	-2.188 to 6.254	No	ns	0.7091
**CD27+, CD62L+**					
**Ctrl** vs. Ctrl 1:1	1.647	-2.574 to 5.868	No	ns	0.8542
**Ctrl** vs. Ctrl 5:1	1.147	-3.074 to 5.368	No	ns	0.965
**Ctrl** vs. **TGFB**	-4.07	-8.291 to 0.1510	No	ns	0.0646
**Ctrl** vs. TGFB 1:1	-0.9467	-5.168 to 3.274	No	ns	0.9849
**Ctrl** vs. TGFB 5:1	-1.45	-5.671 to 2.771	No	ns	0.9091
Ctrl 1:1 vs. Ctrl 5:1	-0.5	-4.721 to 3.721	No	ns	0.9992
Ctrl 1:1 vs. **TGFB**	-5.717	-9.938 to -1.496	Yes	**	0.0027
Ctrl 1:1 vs. TGFB 1:1	-2.593	-6.814 to 1.628	No	ns	0.4609
Ctrl 1:1 vs. TGFB 5:1	-3.097	-7.318 to 1.124	No	ns	0.2671
Ctrl 5:1 vs. **TGFB**	-5.217	-9.438 to -0.9957	Yes	**	0.0076
Ctrl 5:1 vs. TGFB 1:1	-2.093	-6.314 to 2.128	No	ns	0.6833
Ctrl 5:1 vs. TGFB 5:1	-2.597	-6.818 to 1.624	No	ns	0.4595
**TGFB** vs. TGFB 1:1	3.123	-1.098 to 7.344	No	ns	0.2585
**TGFB** vs. TGFB 5:1	2.62	-1.601 to 6.841	No	ns	0.4494
TGFB 1:1 vs. TGFB 5:1	-0.5033	-4.724 to 3.718	No	ns	0.9992

Supplemental Figure 6

Tukey's multiple comparisons test	Mean Diff.	95.00% CI of diff.	Below threshold?	Summary	Adjusted P Value
**CD27+, CD62L-**					
**Ctrl** vs. Ctrl 1:1	8.667	4.446 to 12.89	Yes	****	<0.0001
**Ctrl** vs. Ctrl 5:1	6	1.779 to 10.22	Yes	**	0.0014
**Ctrl** vs. **TGFB**	0.9	-3.321 to 5.121	No	ns	0.9879
**Ctrl** vs. TGFB 1:1	8.333	4.112 to 12.55	Yes	****	<0.0001
**Ctrl** vs. TGFB 5:1	5.267	1.046 to 9.488	Yes	**	0.0069
Ctrl 1:1 vs. Ctrl 5:1	-2.667	-6.888 to 1.554	No	ns	0.4295
Ctrl 1:1 vs. **TGFB**	-7.767	-11.99 to -3.546	Yes	****	<0.0001
Ctrl 1:1 vs. TGFB 1:1	-0.3333	-4.554 to 3.888	No	ns	0.9999
Ctrl 1:1 vs. TGFB 5:1	-3.4	-7.621 to 0.8210	No	ns	0.18
Ctrl 5:1 vs. **TGFB**	-5.1	-9.321 to -0.8790	Yes	**	0.0096
Ctrl 5:1 vs. TGFB 1:1	2.333	-1.888 to 6.554	No	ns	0.5765
Ctrl 5:1 vs. TGFB 5:1	-0.7333	-4.954 to 3.488	No	ns	0.9953
**TGFB** vs. TGFB 1:1	7.433	3.212 to 11.65	Yes	****	<0.0001
**TGFB** vs. TGFB 5:1	4.367	0.1457 to 8.588	Yes	*	0.0387
TGFB 1:1 vs. TGFB 5:1	-3.067	-7.288 to 1.154	No	ns	0.2771

**CD27-, CD62L-**					
**Ctrl** vs. Ctrl 1:1	-11.77	-15.99 to -7.546	Yes	****	<0.0001
**Ctrl** vs. Ctrl 5:1	-9.667	-13.89 to -5.446	Yes	****	<0.0001
**Ctrl** vs. **TGFB**	12.07	7.846 to 16.29	Yes	****	<0.0001
**Ctrl** vs. TGFB 1:1	1.6	-2.621 to 5.821	No	ns	0.8686
**Ctrl** vs. TGFB 5:1	3.2	-1.021 to 7.421	No	ns	0.2348
Ctrl 1:1 vs. Ctrl 5:1	2.1	-2.121 to 6.321	No	ns	0.6804
Ctrl 1:1 vs. **TGFB**	23.83	19.61 to 28.05	Yes	****	<0.0001
Ctrl 1:1 vs. TGFB 1:1	13.37	9.146 to 17.59	Yes	****	<0.0001
Ctrl 1:1 vs. TGFB 5:1	14.97	10.75 to 19.19	Yes	****	<0.0001
Ctrl 5:1 vs. **TGFB**	21.73	17.51 to 25.95	Yes	****	<0.0001
Ctrl 5:1 vs. TGFB 1:1	11.27	7.046 to 15.49	Yes	****	<0.0001
Ctrl 5:1 vs. TGFB 5:1	12.87	8.646 to 17.09	Yes	****	<0.0001
**TGFB** vs. TGFB 1:1	-10.47	-14.69 to -6.246	Yes	****	<0.0001
**TGFB** vs. TGFB 5:1	-8.867	-13.09 to -4.646	Yes	****	<0.0001
TGFB 1:1 vs. TGFB 5:1	1.6	-2.621 to 5.821	No	ns	0.8686

Supplemental Figure 6

Tukey's multiple comparisons test	Mean Diff.	95.00% CI of diff.	Below threshold?	Summary	Adjusted P Value	
**CD57-, KLRG1+**						
**Ctrl** vs. Ctrl 1:1	16.23	11.91 to 20.56	Yes	****	<0.0001	
**Ctrl** vs. Ctrl 5:1	10.53	6.209 to 14.86	Yes	****	<0.0001	
**Ctrl** vs. **TGFB**	45.25	40.92 to 49.57	Yes	****	<0.0001	
**Ctrl** vs. TGFB 1:1	47.12	42.80 to 51.45	Yes	****	<0.0001	
**Ctrl** vs. TGFB 5:1	51.51	47.18 to 55.83	Yes	****	<0.0001	
Ctrl 1:1 vs. Ctrl 5:1	-5.7	-10.02 to -1.376	Yes	**		0.0037
Ctrl 1:1 vs. **TGFB**	29.01	24.69 to 33.34	Yes	****	<0.0001	
Ctrl 1:1 vs. TGFB 1:1	30.89	26.57 to 35.21	Yes	****	<0.0001	
Ctrl 1:1 vs. TGFB 5:1	35.27	30.95 to 39.60	Yes	****	<0.0001	
Ctrl 5:1 vs. **TGFB**	34.71	30.39 to 39.04	Yes	****	<0.0001	
Ctrl 5:1 vs. TGFB 1:1	36.59	32.27 to 40.91	Yes	****	<0.0001	
Ctrl 5:1 vs. TGFB 5:1	40.97	36.65 to 45.30	Yes	****	<0.0001	
**TGFB** vs. TGFB 1:1	1.877	-2.448 to 6.201	No	ns		0.79
**TGFB** vs. TGFB 5:1	6.26	1.936 to 10.58	Yes	**		0.0011
TGFB 1:1 vs. TGFB 5:1	4.383	0.05912 to 8.708	Yes	*		0.0452
**CD57+, KLRG1+**						
**Ctrl** vs. Ctrl 1:1	-0.4667	-4.791 to 3.858	No	ns		0.9995
**Ctrl** vs. Ctrl 5:1	-2	-6.324 to 2.324	No	ns		0.7428
**Ctrl** vs. **TGFB**	9.387	5.062 to 13.71	Yes	****	<0.0001	
**Ctrl** vs. TGFB 1:1	8.16	3.836 to 12.48	Yes	****	<0.0001	
**Ctrl** vs. TGFB 5:1	9.53	5.206 to 13.85	Yes	****	<0.0001	
Ctrl 1:1 vs. Ctrl 5:1	-1.533	-5.858 to 2.791	No	ns		0.8974
Ctrl 1:1 vs. **TGFB**	9.853	5.529 to 14.18	Yes	****	<0.0001	
Ctrl 1:1 vs. TGFB 1:1	8.627	4.302 to 12.95	Yes	****	<0.0001	
Ctrl 1:1 vs. TGFB 5:1	9.997	5.672 to 14.32	Yes	****	<0.0001	
Ctrl 5:1 vs. **TGFB**	11.39	7.062 to 15.71	Yes	****	<0.0001	
Ctrl 5:1 vs. TGFB 1:1	10.16	5.836 to 14.48	Yes	****	<0.0001	
Ctrl 5:1 vs. TGFB 5:1	11.53	7.206 to 15.85	Yes	****	<0.0001	
**TGFB** vs. TGFB 1:1	-1.227	-5.551 to 3.098	No	ns		0.958
**TGFB** vs. TGFB 5:1	0.1433	-4.181 to 4.468	No	ns	>0.9999	
TGFB 1:1 vs. TGFB 5:1	1.37	-2.954 to 5.694	No	ns		0.9339

Supplemental Figure 6

**Tukey's multiple comparisons test**	**Mean Diff.**	**95.00% CI of diff.**	**Below threshold?**	**Summary**	**Adjusted P Value**
**CD57+, KLRG1-**					
**Ctrl** vs. Ctrl 1:1	-5.25	-9.574 to -0.9258	Yes	**	0.0092
**Ctrl** vs. Ctrl 5:1	-4.05	-8.374 to 0.2742	No	ns	0.0783
**Ctrl** vs. **TGFB**	-3.527	-7.851 to 0.7975	No	ns	0.1697
**Ctrl** vs. TGFB 1:1	-8.157	-12.48 to -3.832	Yes	****	<0.0001
**Ctrl** vs. TGFB 5:1	-10.86	-15.18 to -6.532	Yes	****	<0.0001
Ctrl 1:1 vs. Ctrl 5:1	1.2	-3.124 to 5.524	No	ns	0.9617
Ctrl 1:1 vs. **TGFB**	1.723	-2.601 to 6.048	No	ns	0.8428
Ctrl 1:1 vs. TGFB 1:1	-2.907	-7.231 to 1.418	No	ns	0.3602
Ctrl 1:1 vs. TGFB 5:1	-5.607	-9.931 to -1.282	Yes	**	0.0045
Ctrl 5:1 vs. **TGFB**	0.5233	-3.801 to 4.848	No	ns	0.9992
Ctrl 5:1 vs. TGFB 1:1	-4.107	-8.431 to 0.2175	No	ns	0.0715
Ctrl 5:1 vs. TGFB 5:1	-6.807	-11.13 to -2.482	Yes	***	0.0003
**TGFB** vs. TGFB 1:1	-4.63	-8.954 to -0.3058	Yes	*	0.0294
**TGFB** vs. TGFB 5:1	-7.33	-11.65 to -3.006	Yes	***	0.0001
TGFB 1:1 vs. TGFB 5:1	-2.7	-7.024 to 1.624	No	ns	0.4428
**CD57-, KLRG1-**					
**Ctrl** vs. Ctrl 1:1	-10.47	-14.79 to -6.142	Yes	****	<0.0001
**Ctrl** vs. Ctrl 5:1	-4.467	-8.791 to -0.1425	Yes	*	0.0392
**Ctrl** vs. **TGFB**	-51.07	-55.39 to -46.74	Yes	****	<0.0001
**Ctrl** vs. TGFB 1:1	-47.13	-51.46 to -42.81	Yes	****	<0.0001
**Ctrl** vs. TGFB 5:1	-50.2	-54.52 to -45.88	Yes	****	<0.0001
Ctrl 1:1 vs. Ctrl 5:1	6	1.676 to 10.32	Yes	**	0.002
Ctrl 1:1 vs. **TGFB**	-40.6	-44.92 to -36.28	Yes	****	<0.0001
Ctrl 1:1 vs. TGFB 1:1	-36.67	-40.99 to -32.34	Yes	****	<0.0001
Ctrl 1:1 vs. TGFB 5:1	-39.73	-44.06 to -35.41	Yes	****	<0.0001
Ctrl 5:1 vs. **TGFB**	-46.6	-50.92 to -42.28	Yes	****	<0.0001
Ctrl 5:1 vs. TGFB 1:1	-42.67	-46.99 to -38.34	Yes	****	<0.0001
Ctrl 5:1 vs. TGFB 5:1	-45.73	-50.06 to -41.41	Yes	****	<0.0001
**TGFB** vs. TGFB 1:1	3.933	-0.3909 to 8.258	No	ns	0.094
**TGFB** vs. TGFB 5:1	0.8667	-3.458 to 5.191	No	ns	0.9909
TGFB 1:1 vs. TGFB 5:1	-3.067	-7.391 to 1.258	No	ns	0.3022
